# Molecular design of MRI probes for targeting amyloid-β species: from *in vitro* binding to *in vivo* imaging

**DOI:** 10.7150/thno.131311

**Published:** 2026-06-04

**Authors:** Yupeng Shi, Ruiyang Zhang, Miaoqing Li, Yaning Xia, Mengyang Zhou, Rui Cao, Qing Zhou, Yong Zhang

**Affiliations:** Department of MRI, The First Affiliated Hospital of Zhengzhou University; Henan Key Laboratory of Functional Magnetic Resonance Imaging and Molecular Imaging, Zhengzhou 450052, China.

**Keywords:** Amyloid-β, MRI, Molecular imaging, Alzheimer's disease, Nanoprobe

## Abstract

The aberrant aggregation of amyloid-β (Aβ) is a central pathological marker of Alzheimer's disease (AD) and shows different neurotoxic properties in various forms, such as monomers, oligomers, fibers and plaques. In recent years, great progress has been achieved in the molecular design and the development of magnetic resonance imaging (MRI) probes targeting Aβ species. They provide powerful tools for the early diagnosis and pathological investigation of AD. Here, we systematically review the molecular design strategies and recent advances in Aβ-targeted MRI probes. First, we introduce the molecular pathological basis of Aβ aggregation and the importance of Aβ as an imaging target. Second, we detail the core components of probe design, including the selection of targeting ligands (e.g., peptide mimetics, small molecules, and antibody fragments), optimization of signal units (e.g., Gd(III), Mn(II), superparamagnetic iron oxide nanoparticles (SPIONs), and ¹⁹F), and the delivery strategies to enhance blood-brain barrier (BBB) penetration. We focus on how the probes achieve the transition from high-affinity binding *in vitro* to high-contrast imaging *in vivo* by means of changes in proton relaxation times (T1/T2) or the chemical exchange saturation transfer (CEST) effect upon binding to Aβ. Furthermore, the imaging performance of various probes (small molecule probes, nanoprobes, and smart responsive probes) in transgenic AD models is compared and evaluated, and the challenges related to sensitivity, specificity, and biosafety are discussed. Finally, we discuss future directions for Aβ-targeted MRI probes, including oligomer-specific probes, multimodal imaging probes, and theranostic platforms that include both diagnostic and therapeutic functions. Through interdisciplinary innovation in molecular design, the next generation of MRI probes is expected to play a key role in preclinical research, early diagnosis, and therapeutic evaluation of AD.

## 1. Introduction

Alzheimer's disease (AD) is the most common neurodegenerative disorder and the leading cause of dementia worldwide. The characteristic of the disease is the gradual deterioration of cognitive functions due to damage to neural connections, which may lead to the death of many elderly people 1, 2. As the population ages, AD has become a serious global health problem. Currently, more than 55 million people suffer from memory disorders, most of them suffer from Alzheimer's disease. This number is expected to double by 2050, placing a significant burden on medical systems and the economy. At the molecular level, the amyloid-β (Aβ) cascade remains the main pathway to understand the mechanism of the onset of Alzheimer's disease. In this pathway, the abnormal cleavage of the amyloid precursor protein (APP) leads to a series of reactions that promote the accumulation of Aβ peptide. These peptides are initially present in the form of soluble monomers, then aggregate to form oligomers and protofibrils, and end up forming insoluble plaques within the brain 3, 4. The main difference lies in the relative neurotoxicity of each form of Aβ, as well as its effect on disease progression. Distinguishing these species is therefore critical in molecular imaging. Soluble Aβ oligomers, although relatively low in abundance, have significantly more synaptotoxicity than fibrillar plaques and show a stronger correlation with the severity of cognitive impairment in AD 5. On the other hand, protofibrils are considered highly pathogenic intermediate aggregates with strong seeding and propagation potential, while mature fibrils and plaques mainly reflect the cumulative amyloid burden and later-stage pathological deposition 6, 7. These findings show that it is important to develop non-invasive imaging methodologies that can detect and discriminate Aβ species in the living brain.

For this purpose, several imaging modalities have been extensively studied. Positron emission tomography (PET) with radiolabeled tracers such as [¹¹C]PiB (Pittsburgh Compound B) and its [¹⁸F]-labeled derivatives (e.g., [¹⁸F]florbetapir, [¹⁸F]flutemetamol, [¹⁸F]florbetaben) allows the *in vivo* visualization of fibrillar Aβ plaques and has been incorporated into the revised diagnostic criteria for AD 8-11. The above advances allow the *in vivo* visualization of Aβ plaque distribution and have been included in the updated diagnostic criteria for AD. However, PET imaging has inherent limitations. The required radiation exposure limits safe longitudinal applications, particularly in presymptomatic studies 12. Spatial resolution is limited to ~3-5 mm, which is insufficient for detailed anatomical localization 13,14. High cost and complex tracer production also limit accessibility. Most importantly, available PET probes target only the insoluble fibrillar aggregates and lack adequate sensitivity and specificity for the conformationally dynamic soluble oligomers, the Aβ species most closely related to synaptic dysfunction and disease progression 15,16.

Magnetic resonance imaging (MRI) provides a compelling alternative for patients who cannot rely on nuclear medical imaging techniques. Unlike PET, MRI does not involve exposure to ionizing radiation, and offers good soft tissue contrast, high spatial resolution, and multi-parameter signal analysis capability. Thanks to these properties, MRI is particularly suitable for frequent and non-invasive evaluation of disease progression or treatment response 17, 18. However, the ability to distinguish between Aβ accumulation and surrounding brain tissue may be limited in standard MRI sequences, largely because of their signaling properties. To overcome this major limitation, scientists have created molecular probes that target the protein. These probes are designed to specifically bind to certain forms of Aβ, allowing them to cross the blood-brain barrier and cause measurable changes in the MRI signal. These changes typically arise from altered proton relaxation behavior, including changes in T1, T2, or T2* relaxation, or through chemical exchange saturation transfer (CEST) mechanisms. This molecular imaging approach poses very challenging design issues, as it requires high target specificity, efficient signal amplification, good pharmacokinetics, and excellent biocompatibility. The rational design of Aβ-targeted MRI probes is an interdisciplinary endeavor involving chemical synthesis, bioconjugation strategies, nanomaterial engineering, and *in vivo* validation. In recent years, diverse probe architectures have been obtained, from small molecule paramagnetic complexes to functionalized nanoparticles and activatable CEST agents. All these probes need to be evaluated in a proper translational pipeline from *in vitro* binding assays and selectivity profiling to *in vivo* imaging efficacy and safety assessment in preclinical models.

**Figure 1:**
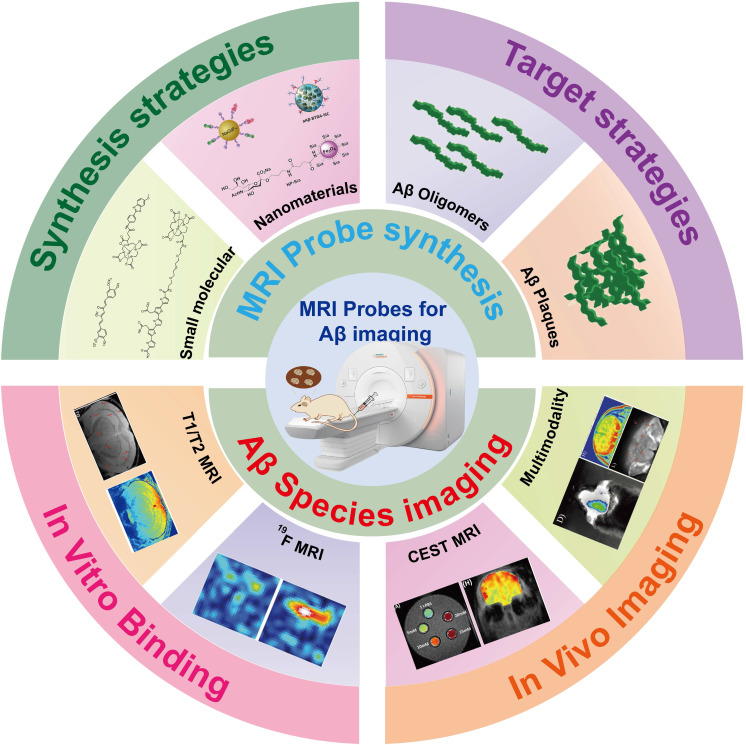
Schematic illustration of synthesis, functionalization and applications of Aβ-targeted MRI probes. Aβ-targeted MRI probes are synthesized from small molecules or nanomaterials that then functionalized to target Aβ oligomers and plaques. Their performance is validated by *in vitro* binding assays and *in vivo* imaging for Alzheimer’s disease diagnosis.

Here, we systematically review the molecular design principles and recent progress made in the development of MRI probes capable of targeting cerebral Aβ species, with special attention to the gap between *in vitro* characterization and *in vivo* imaging application. We assess the design strategies that have been proposed for different categories of probes, such as gadolinium- and manganese-based T1 agents, superparamagnetic iron oxide nanoparticles (SPIONs), fluorine-19 (¹⁹F) probes, and CEST-based sensors. We pay particular attention to new approaches for the selective detection of soluble Aβ oligomers (**Figure 1**). Moreover, we report the preclinical imaging results and discuss the remaining problems related to sensitivity, specificity, pharmacokinetics, and clinical translation. Finally, we give a perspective on the development of the next generation of theranostic probes and multimodal imaging platforms. This study aims to promote future developments in the molecular design of MRI probes and accelerate their transformation into useful clinical tools for early diagnosis and management of AD through a comprehensive and balanced review.

## 2. Design Principles for Aβ-Targeted MRI Probes

Constructing an effective MRI probe for imaging Aβ in the brain requires precise alignment between the biological properties of the target, contrast composition, and pharmacodynamic modulation. The following explains some of the basic design principles when logically developing this probe. This paper will focus in particular on target selection, key molecular components, brain barrier bypass strategy, as well as methodology for improving probe behavior and safety at the same time.

### 2.1. Pathogenic Role of Aβ and Rationale for Molecular Imaging

**Figure 2:**
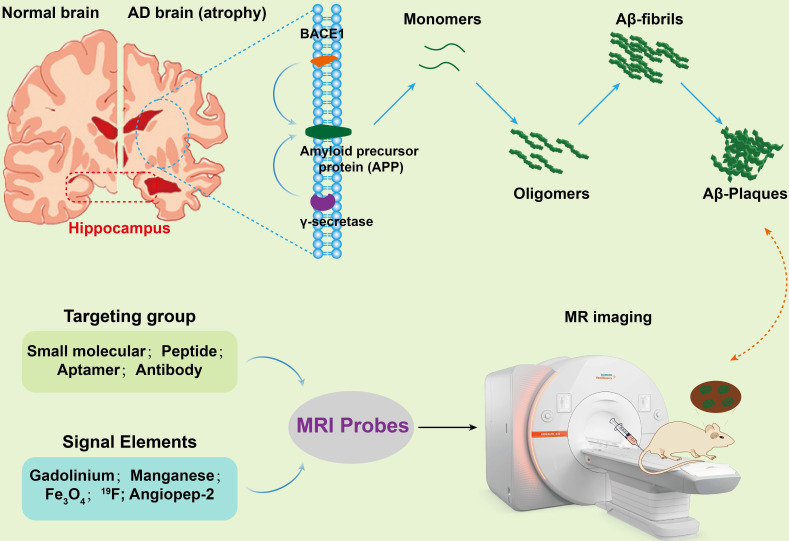
Schematic depiction of the mechanism involved in the detection of Aβ species with MRI probes. In AD brain, BACE1 and γ-secretase cleave APP to generate Aβ monomers, which aggregate into oligomers, fibrils, and finally Aβ plaques. MRI probes combine a targeting group (small molecule, peptide, aptamer, or antibody) with a signal element (gadolinium, manganese, Fe_3_O_4_, ¹⁹F, or Angiopep-2) to specifically bind Aβ species and enable MR imaging of amyloid pathology.

The accumulation of amyloid-β (Aβ) is a major factor in the harmful changes associated with Alzheimer's disease. According to the amyloid cascade hypothesis, Aβ is believed to result from a series of amyloid precursor protein (APP) breakdowns. This process is regulated by the enzymes BACE1 and γ-secretase, which play an important role in the amyloid production pathway (see **Figure 2**). Cleavage of APP by γ-secretase leads to the formation of Aβ monomers of different lengths, among which Aβ42 is critical, as it has a greater degree of hydrophobicity and tendency to aggregate compared to Aβ40. Structurally, the Aβ monomer consists of a hydrophilic N-terminus and a hydrophobic C-terminus, facilitating self-aggregation in an aqueous environment. Thus, insoluble protofibrils or fibrils are formed as a result of self-assembly, and ultimately transform into insoluble amyloid plaques. Aβ_42_ is also more easily aggregated than Aβ40, because it has two additional hydrophobic residues at its C-terminus, and is one of the main components of senile plaques and vascular deposits within the brain. In addition to direct neurotoxicity, Aβ deposition also stimulates a series of secondary pathological reactions such as neuroinflammation, oxidative stress, and abnormal phosphorylation of tau protein. Since Aβ aggregates play a key role in this process, the specific and accurate detection of Aβ species in living brain tissue, especially soluble oligomers and fibrillar plaques, is an important goal in diagnostics and scientific research.

### 2.2. Strategic Target Selection

#### 2.2.1. Insoluble Aβ Plaques

Insoluble Aβ plaques are a typical pathological feature of Alzheimer's disease and are the primary target in the development of MRI tests 19,20. These deposits contain a rich content of cross-β sheets, forming stable hydrophobic binding pockets that absorb small organic molecules 21. Initial probe designs relied primarily on thioflavin T derivatives and biphenyl ethylene alternatives, which bind to beta-sheet regions via π-π stacking and hydrophobic interactions 22. This method has been widely adopted due to the abundance of these plaques and their structural stability, as well as the availability of abundant pharmacological data based on PET tracers previously used as targets for Aβ 23. However, recent clinical evidence suggests that the relationship between plaques and loss of cognitive function is only moderate, and that plaque deposition occurs at a relatively late stage of the disease, making it unsuitable for diagnosis before the onset of symptoms 24.

#### 2.2.2. Soluble Aβ Oligomers

The focus has recently turned toward soluble Aβ oligomers (oAβ), which are highly neurotoxic, although they have low abundance physiologically. Unlike fibrillar aggregates, oligomers are structurally heterogeneous, dynamic, and lack conserved binding epitopes 19. To design oligomer-selective probes, we need excellent conformational discrimination. The strategies that are being pursued are: (i) monoclonal antibody fragments against oligomer-specific epitopes 25; (ii) rationally designed constrained cyclic peptides; and (iii) smart scaffolds that are able to perform conformation-dependent switching of the signal. Success in this direction would allow prodromal detection and therapy monitoring and would represent a paradigm shift in AD management.

### 2.3. Core Molecular Constituents

The design of core molecular constituents of Aβ-targeted MRI probes is in line with the principle of centering on the pathological aggregate characteristics of Aβ, balancing targeting specificity/affinity, MRI signal detectability, and compatibility with other probe modules. Selection of each component is driven by imaging demands and constrained by physicochemical properties. Molecular structural optimization is employed to achieve an optimal balance between binding performance and imaging efficacy.

#### 2.3.1. Targeting Moieties

Targeting moieties confer probe specificity and binding affinity via precise molecular recognition. Ligands for PET imaging are developed through structural optimization of well-validated Aβ-PET tracers (e.g., PiB, AV-45), where radionuclides are replaced with MRI-active complexes 26, 27. This design strategy leverages mature high-affinity molecular frameworks, but the relatively bulky MRI module tends to raise lipophilicity and compromise blood-brain barrier penetration. Accordingly, the introduction of hydrophilic linkers such as PEG and trilysine is essential to separate the chelate from the targeting core; omitting this structural modification can result in a one-order-of-magnitude decline in binding affinity. Aβ-mimetic peptides (e.g., KLVFF) are biocompatible domains that bind to aggregates by self-recognition. Their design is based on β-sheet complementarity, but linear peptides suffer from poor metabolic stability; it is usually necessary to cyclize or modify the backbone to improve *in vivo* performance 28. *De novo* designed ligands (e.g., aptamers, phage-display peptides) allow the identification of novel scaffolds with better aggregation-state selectivity 29-31. The design rationale is to target conformationally dynamic epitopes, particularly on toxic oligomers, which are not accessible to conventional ligands. For example, the DNA aptamer ob5 binds exclusively to Aβ oligomers, and when conjugated to Gd-DOTA, it produces rapid MRI enhancement in early-stage AD mice 32.

#### 2.3.2. Signal Elements

The signal transduction module converts molecular binding events into detectable MRI contrast by several distinct mechanisms: (1) T1 contrast agents (Gd³⁺, Mn²⁺ complexes) shorten longitudinal relaxation time to generate positive contrast. The design aims to increase relaxivity (r1) by rigidification of the chelate or conjugation to large carriers, because slower molecular tumbling upon Aβ binding amplifies r1 by 2- to 4-fold 33, 34. Macrocyclic chelating agents such as DOTA and DO3A are essential to guarantee the biosafety of loaded metal ions. Mn²⁺ has higher inherent relaxivity, but the signal amplification triggered by target binding is far weaker (only 2-3 fold, versus 3-5 fold for Gd³⁺). It also tends to bind nonspecifically with transferring, which greatly restricts its clinical translation 35, 36. (2) T2/T2* contrast agents (mainly SPIONs) disrupt the uniformity of the local magnetic field, generating high-sensitivity negative contrast signals. Their design mainly relies on surface modification with targeting ligands (e.g., curcumin, antibodies) and biocompatible coatings (e.g., PEG, PLA) to boost blood-brain barrier penetration and reduce uptake by the reticuloendothelial system. Thanks to their ultrahigh r2 relaxivity (50-200 mM⁻¹ s⁻¹), these agents can be detected at low iron concentrations, yet their negative contrast is easily misdiagnosed as cerebral hemorrhage 37. (3) ¹⁹F-based probes enable quantitative MRI imaging due to the almost no endogenous fluorine background *in vivo*. The core difficulty in their design is to avoid signal attenuation after target binding. Hydrophobic fluorinated probes often get sequestered in myelin and lose detectable signals; introducing flexible PEG spacers can separate the ¹⁹F module from the rigid targeting structure, keeping a clear NMR signal peak even after binding 38. To achieve oligomer selectivity, conformational locking strategies (e.g., stabilizing the flexible keto form of curcumin) allow preferential binding to loosen Aβ oligomers rather than mature fibrils 39. However, these probes require high injection doses (100–200 mg/kg) and can only be applied under high-field MRI (≥ 7 T). (4) CEST agents work by transferring magnetization from exchangeable protons (-OH, -NH) to bulk water molecules. Effective design requires a suitable proton exchange rate (kₑₓ = 10-1000 s^-1^) and a distinguishable chemical shift offset (> 1 ppm) from water. The repurposed PET tracer PiB contains a phenolic hydroxyl group that produces a CEST signal at 5.8 ppm, supporting label-free imaging of amyloid plaques 40. Angiopep-2 peptide, which crosses the BBB through LRP1-mediated transport, shows a CEST peak at 3.2 ppm; its signal intensity increases linearly with age in AD model mice, supporting long-term longitudinal disease monitoring 41, 42. The biggest drawback of CEST agents is low detection sensitivity, which calls for ultra-high-field MRI equipment (7 T or 9.4 T).

### 2.4. Blood-Brain Barrier Penetration Strategies

**Figure 3:**
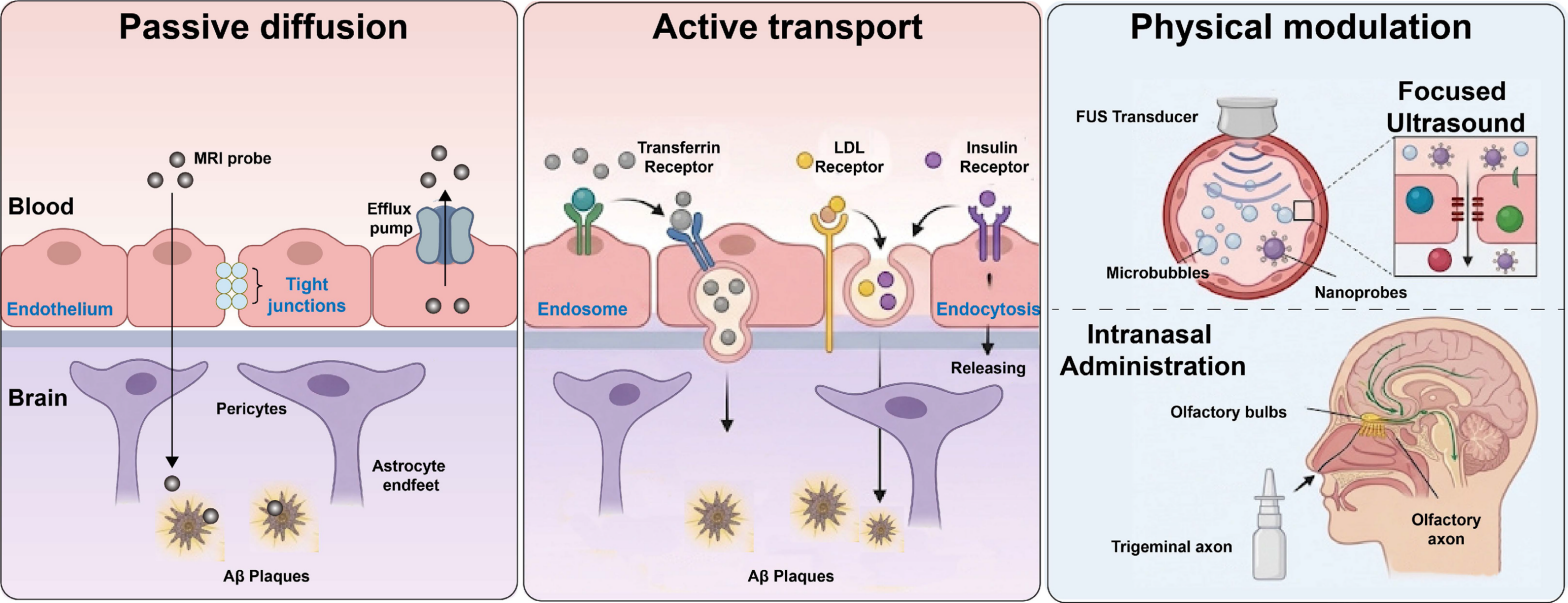
Schematic diagram of blood-brain barrier penetration strategies for Aβ-targeted MRI probes. Three main strategies enable Aβ-targeted MRI probes to cross the blood-brain barrier (BBB). Passive diffusion is limited by efflux pumps but can be locally enhanced by Aβ plaques. Active transport relies on receptor-mediated transcytosis (e.g., transferrin receptor) across the endothelium. Physical modulation includes focused ultrasound with microbubbles to reversibly open tight junctions, or intranasal administration that bypasses the BBB via olfactory/trigeminal pathways.

Rational strategies to enhance blood-brain barrier penetration are essential for developing effective MRI probes targeting Aβ. Such strategies can be broadly classified into three mechanistic categories (**Figure 3**): physicochemical optimization for passive diffusion; active transport exploiting endogenous receptor systems; and physical modulation for forced permeability enhancement 43, 44. For small-molecule probes, physicochemical optimization is the main approach: it is mandatory to respect Lipinski's Rule of Five parameters-molecular mass <500 Da, calculated log P of 1-3, and fewer than five hydrogen bond donors-to allow efficient passive diffusion across endothelial membranes 45. When passive diffusion is kinetically or thermodynamically constrained, as for larger molecular constructs, active transport via receptor-mediated transcytosis can be an alternative; this strategy consists of conjugation to ligands targeting endogenous BBB transport systems, including the transferrin receptor, insulin receptor, and LDL (low-density lipoprotein) receptor-related protein, in order to exploit physiological nutrient delivery pathways for CNS access 46-48. For macromolecular or nanoparticulate systems that are not compatible with the above molecular translocation routes, physical modulation techniques allow bypassing of the BBB by methods such as focused ultrasound with microbubble cavitation to achieve transient, localized barrier disruption, or intranasal administration that exploits the olfactory-cerebral axis for direct brain delivery. These invasive methods show promising preclinical results, but before moving to clinical application, a comprehensive safety assessment, repeated verification, and standardization of criteria are required 49,50. The most appropriate delivery strategy largely depends on the physical and chemical properties of the probe. Physicochemical optimization is suitable for small molecular probes, while active transport fits larger structures such as antibody conjugates, and physical methods are used in cases where nanoparticles or strongly hydrophobic optimization are required. For each delivery method there is an inherent compromise between delivery efficiency, target specificity, and practical feasibility. When rationally designing Aβ-targeted MRI probes, these factors must be carefully considered.

### 2.5. Pharmacokinetic and Safety Profiles

The feasibility of using Aβ-targeted MRI probes in clinical environments depends largely on a comprehensive assessment of their overall pharmacokinetic and safety properties. Thus, intracerebral distribution, systemic clearance, and biocompatibility should be systematically improved 18,51,52. From the perspective of brain pharmacokinetics, designing an effective probe requires precise adjustment of its dynamic properties. The interaction between the probe and the target must have sufficient duration to successfully obtain images. At the same time, unbound particles should be removed quickly, minimizing the impact of non-specific background signals on image results. The designed probe should show stability when bound to Aβ aggregates, while non-bound molecules should be easily removed from brain tissue. Effective systemic clearance requires rapid excretion through the liver, bile, or kidney. This process leads to a decrease in background signals in the blood, reducing toxic effects on the body. This is especially important for probes containing heavy metals such as gadolinium or manganese. If these metals remain in the body for a long time, they can accumulate in tissues, leading to health problems such as gadolinium-induced systemic fibrosis or manganese-induced encephalopathy. The concept of biocompatibility is not limited to just the basic toxicity of metals. It also includes multiple safety aspects: the thermodynamic and kinetic stability of metal-chelate complexes in physiological environments; the immunogenicity of nanomaterial-based components; complete metabolic breakdown of all molecular parts and their metabolites; as well as their final excretion routes. Developing MRI probes for Aβ targeting involves multi-parameter optimization, covering molecular recognition, contrast production, blood-brain barrier crossing, pharmacokinetic behavior, and biosafety. Targeting strategies focused on amyloid plaques are technically well-established, yet their diagnostic utility is limited to the advanced stages of Alzheimer's disease. By contrast, strategies targeting Aβ oligomers offer great promise for early disease intervention, though they present significant design hurdles related to conformational selectivity. The selection of signal amplification systems and BBB-crossing strategies should be tailored to specific imaging goals and the inherent properties of the probe itself. Safety evaluation should be integrated throughout every stage of probe development. With continuous optimization of these design guidelines, next-generation Aβ-targeted MRI probes will make important contributions to the early diagnosis, patient stratification, and therapeutic efficacy assessment of Alzheimer's disease.

## 3. Recent Advances in Various Aβ-Targeted MRI Probes

Given the critical importance of early diagnosis and rapid intervention in Alzheimer’s disease (AD), the development of non-invasive imaging technologies that can specifically detect Aβ accumulation in the brain is becoming increasingly important. In recent years, significant progress has been made in the study of molecular probes based on MRI. MRI has several advantages over PET. Due to the absence of ionizing radiation, it has a higher spatial resolution and can provide detailed anatomical information. However, there are also problems with the development of MRI probes that effectively target Aβ. The main challenges are passing through the blood-brain barrier (BBB), the ability to detect with sufficient sensitivity, and how to accurately identify the target. In this section, we systematically summarize the latest scientific trends regarding different types of Aβ MRI probes, from small molecules to nanoparticle systems, from traditional imaging mechanisms to new strategies, and highlight the latest achievements and general development trends in this area.

### 3.1 Small molecular probe

**Figure 4:**
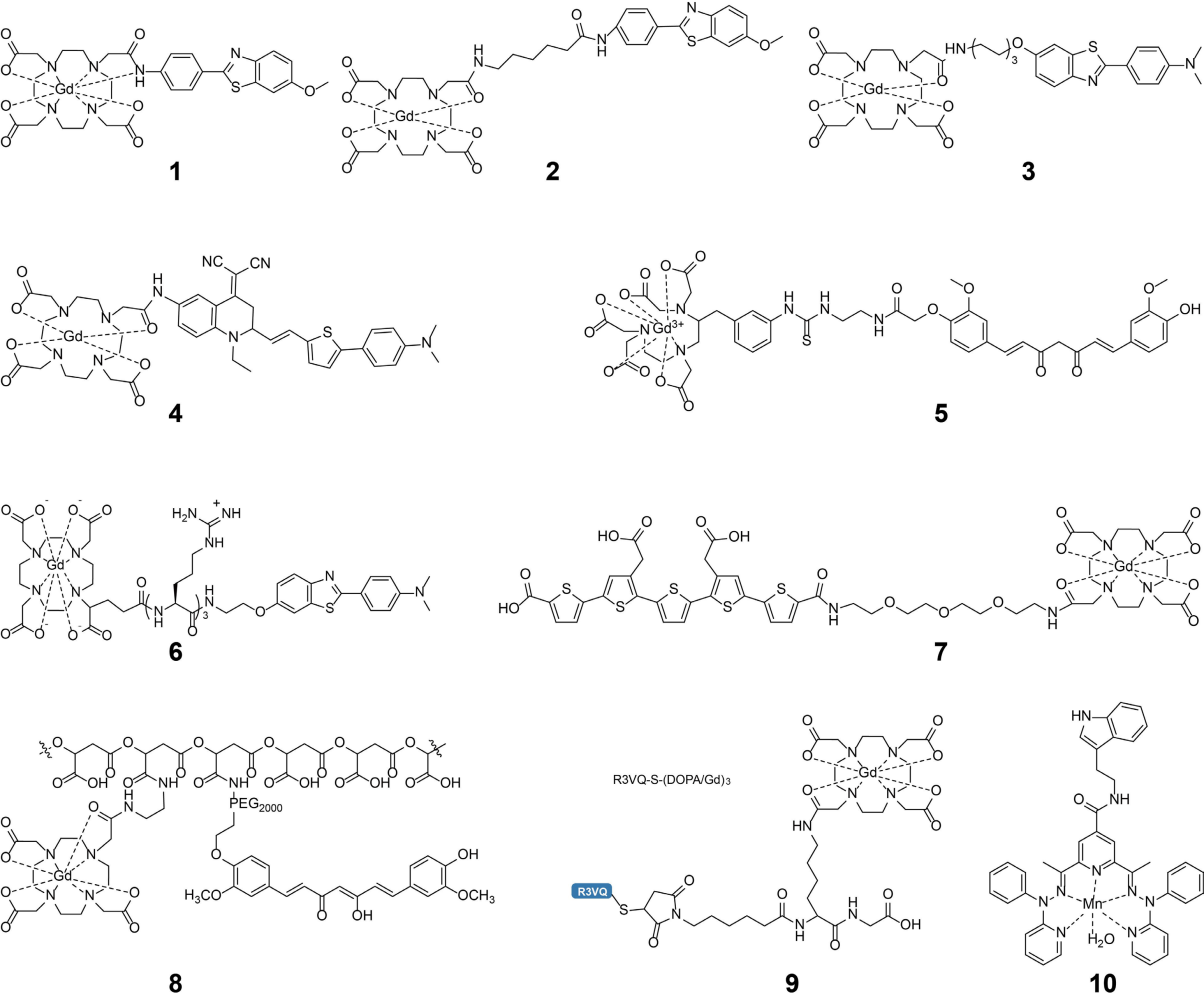
Chemical structures of gadolinium or manganese complexes for MRI probe.

In particular, small-molecule probes, which do not require nanocarriers and connect target moieties to the signal unit via a common linker, are a central topic of research in Aβ MRI probes. These probes have a relatively low molecular weight (less than 1000 Da) and good blood-brain barrier permeability. In T1-enhanced imaging, the positive contrast is observed mainly due to the reduced T1 relaxation time of neighboring water molecules, which is caused by the presence of a small molecular probe. These probes use various signal-generating elements such as Gd³⁺, Mn²⁺, and other paramagnetic ions, which can further reduce the T1 relaxation time of their surrounding water molecules. This leads to a stronger signal, or positive contrast, in T1-weighted images (**Figure 4**). Alternatively, ¹⁹F-based probes offer another way to image Aβ. Unlike T1 agents, they are detected directly by ¹⁹F MRI, so there’s no background signal from water in the brain. That makes it easy to spot Aβ deposits. These probes are also small and can cross the blood-brain barrier. Their signal depends only on probe concentration, which means they can give a quantitative readout of Aβ burden.** Table 1** summarizes representative small-molecule MRI probes for Aβ imaging.

#### 3.1.1. Gd(III)-Based Probes

Thanks to their paramagnetic property that shortens the relaxation time of water protons, Gd(III)-based probes are now the most widely studied contrast agents in AD MRI research. The main design approach relies on the use of chelators that bind gadolinium (Gd) to the Aβ target ligand, with the aim of improving its absorption across the blood-brain barrier (BBB) and binding with Aβ simultaneously. However, there were several challenges in this approach. In the initial studies of Poduslo et al., a modified peptide of Aβ that binds to Gd-DTPA chelators was developed. This probe showed a better ability to cross BBB, as well as being able to target amyloid plaques in mice with genetic recombination after intravenous injection, successfully validating the concept of peptide-based MRI probes 53. The initial design of this molecular probe was very simple, as classical fibril-based Aβ ligands (e.g. benzothiazole (BTA), benzazole, and PiB derivatives) already tested in positron emission imaging (PET) studies were combined with clinically applicable gadolinium chelators (e.g. DOTA and DO3A) 54-56. In 2014, Berth et al. studied a revolutionary system using a built-in module strategy, in which 16 different types of probes were manufactured. They systematically studied how the chelator charge and the linker charge (positive or negative) affect the performance of the probe, by analyzing the impact of both on the performance of the probe. The results showed that positively charged linkers (e.g. trilysine/tropizine) significantly increase the ability to bind to Aβ_1-42_ by separating the target ligand from negative chelators and reducing the strength of electrostatic repulsion, and that some probes significantly increase their relaxivity and stability in serum (r₁ up to 29.3 mM⁻¹ s⁻¹), but their BBB absorption efficiency remained relatively low. In an *in vitro* BBB model, the brain concentration of these probes is only 0.1%-0.5% of their plasma concentration, a major bottleneck limiting *in vivo* application. The work of Martins et al. (2013, 2014) next introduced a “smart” responsive mechanism. They developed the Gd(DO3A-PiB) probe, which exhibits environmentally responsive relaxivity enhancement: when the probe binds to Aβ aggregates or human serum albumin, the molecular rotational motion is restricted, leading to a 2- to 4-fold increase in r₁ 33, 34. This “activatable” contrast enhancement helps improve the signal-to-noise ratio between target and non-target areas. They also explored the possibility of constructing multimodal (MRI/SPECT) probes based on the same PiB-DO3A scaffold, providing a conceptual framework for cross-modal validation of imaging specificity.

**Figure 5:**
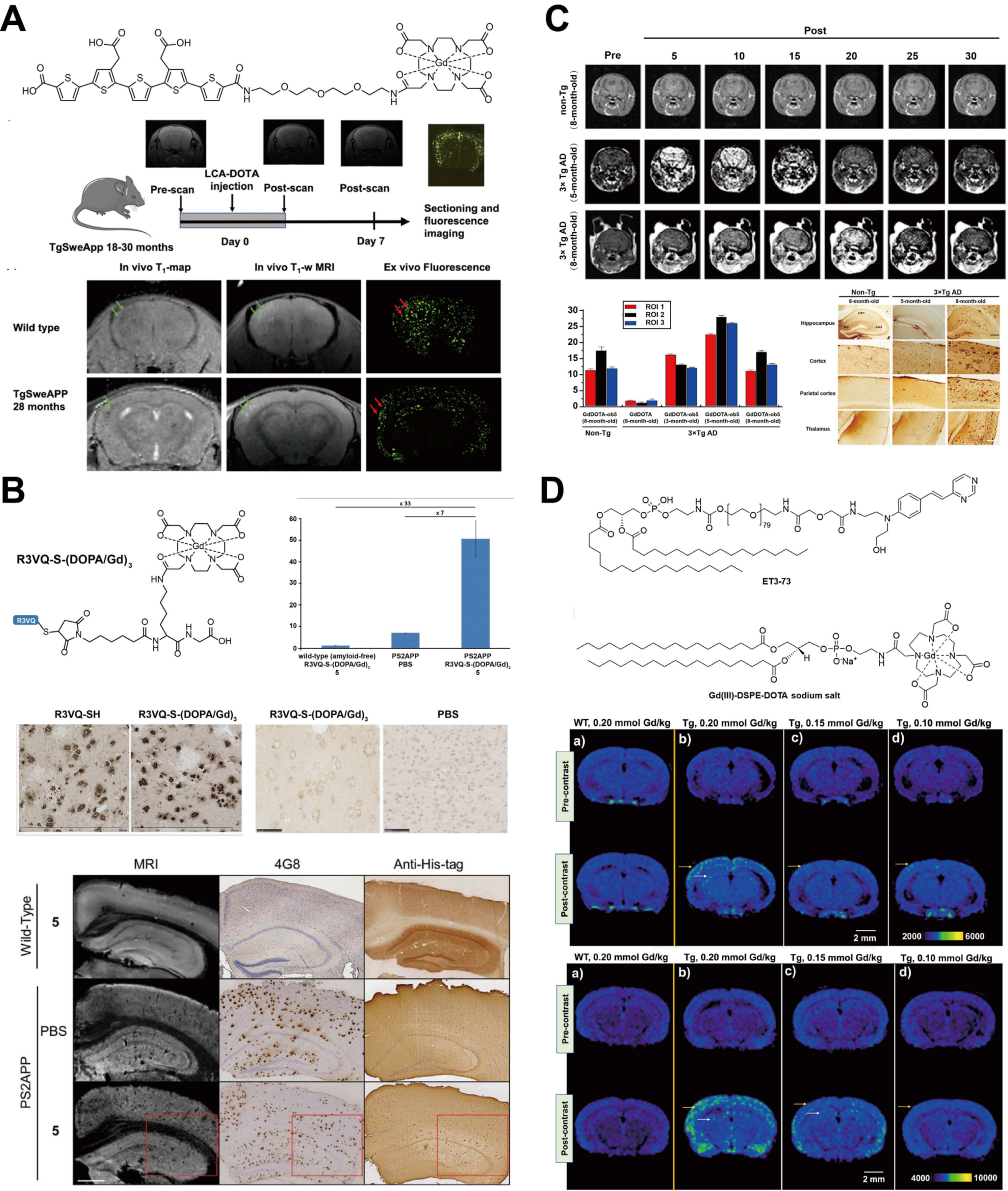
**Gd-based small molecule MR probes used for Aβ species imaging.** (A) The imaging protocol of TgSweAPP mice using the probe LCO-DOTA, as well as the T1-mapping, T1WI, and confocal microscopy imaging of wild-type (WT) and TgSweAPP mice in the same brain section. Adapted with permission from^45^, Copyright 2023, SAGE. (B) Signal enhancement after injection of the GdDOTA-ob5 contrast agent and the corresponding immunohistological on non-Tg and 3xTg AD mice of different ages. Adapted with permission from 58, Copyright 2017, Taylor & Francis. (C) Targeted binding of R3VQ-S-(DOTA/Gd)_3_, BBB permeability and *in vivo* T1-weighted MR images of transgenic AD model mice. Adapted with permission from 32, Copyright 2023, Wiley-VCH. (D) The T1WI-SE and FSE-IR imaging comparison of ADx-001 (consisting of ET3-73 and Gd(III)-DSPE-DOTA) in transgenic mice (Tg APPswe/PSEN1dE9) and WT mice. Adapted with permission from^60^, Copyright 2020, Springer Nature.

Although the above-mentioned first-generation probes based on traditional small-molecule ligands made some progress in affinity optimization, the problem of BBB penetration remained unsolved. Therefore, the second-generation design strategy was to integrate targeting modules with intrinsic BBB-penetrating ability, achieving “targeting-delivery” integration. The research by Sulheim et al. (2023) exemplifies this direction. They used luminescent conjugated oligothiophene (LCO) as a multifunctional unit. On one hand, it specifically recognizes conformational epitopes on Aβ aggregates via hydrophobic interactions; on the other hand, it effectively facilitates probe transcytosis across a brain microvascular endothelial cell monolayer by adsorption-mediated transcytosis (with 2.3-fold higher efficiency than free Gd-DOTA) 45 (**Figure 5A**). The LCO-DOTA-Gd probe enabled long-lasting, high-contrast imaging in 28-month-old transgenic AD mice, and the T₁ signal intensity was highly correlated with cortical plaque burden (R^2^ = 0.7), demonstrating the possibility of longitudinal monitoring of disease progression.

In order to achieve higher targeting specificity and affinity and to partially compensate for the difficulty of using antibody-based large molecules to cross the BBB, researchers began to use smaller-sized, biologically derived high-affinity ligands. Vandesquille et al. (2017) 58 were the first to use an extremely low molecular weight (~15 kDa) camelid-derived single-domain antibody (variable domain of the heavy chain of a heavy-chain antibody, VHH) in the construction of Gd-based MRI probes. They compared random conjugation with site-specific conjugation strategies. They found that introducing a cysteine at the C-terminus of the VHH for specific linkage to maleimide-modified trimeric Gd-DOTA yielded the chemically homogeneous probe R3VQ-S-(DOTA/Gd)_3_. This probe perfectly retains the high affinity of the VHH for Aβ (IC_50_ = 19 nM for Aβ_40_), and its small size enables spontaneous BBB penetration. More importantly, due to increased molecular rigidity after conjugation and the ability of the VHH surface to capture more water molecules, this probe exhibits excellent relaxivity at every field strength, with its r₁ value up to 10 times that of the clinical probe DOTAREM (35 mM⁻¹ s⁻¹), giving excellent contrast in *ex vivo* brain tissue imaging. Another high-specificity strategy comes from nucleic acid aptamer technology (**Figure 5B**). Kim et al. (2023) 32 used the Systematic Evolution of Ligands by Exponential Enrichment (SELEX) technique to screen a DNA aptamer, ob5, that recognizes oAβ, and constructed the GdDOTA-ob5 probe (**Figure 5C**). The important feature of this probe is its excellent selectivity: it reacts only with neurotoxic oAβ and shows almost no reaction with monomeric Aβ. In a 5-month-old 3xTg-AD mouse (early stage of oAβ deposition), rapid MRI signal enhancement was observed just 5 minutes after injection, and the fluorescence imaging signal intensity was much higher than in other control groups, making this a very promising tool for ultra-early diagnosis of AD.

Besides modifying the probe molecules themselves, using nanocarrier systems to optimize probe pharmacokinetics and relaxivity is also an important direction. Patil et al. used the biocompatible polymer poly(malic acid) (PMLA) as a nanocarrier and covalently linked the natural targeting molecule curcumin (CUR) and Gd-DOTA to construct the PMLA-CUR-Gd-DOTA probe, achieving specific labeling and relaxivity enhancement of Aβ plaques 59. Badachhape et al. made a more structurally complex, PEGylated liposomal probe, ADx-001 60. This probe displays the targeting molecule ET3-73 on the liposome surface and encapsulates Gd-DOTA inside. In APP/PSEN1 transgenic mouse models, this probe showed 100% imaging specificity, with sensitivity >80% at a high dose (0.20 mmol Gd/kg), and biosafety assessments indicated very low toxicity. For this type of nanocarrier-based macromolecular probe (particle size ~140 nm), BBB penetration mainly depends on disruption of BBB integrity in later disease stages or slow permeation via the peripheral clearance system; it may not be suitable for detecting the earliest stage of Aβ pathological changes where the BBB is mostly intact (**Figure 5D**).

The field of Aβ protein imaging has made great progress with small-molecule Gd-based MRI probes. Their design philosophy has evolved from initial exploration of structure-activity relationships to integration of functional modules, and now to the use of biological tools such as nanobodies and aptamers to achieve ultra-high specificity and selectivity. The most advanced probes have achieved excellent performance in relaxation efficiency (r1 > 25 mM⁻¹ s⁻¹), targeting affinity (IC₅₀ at nanomolar level), and precise recognition of pathogenic oAβ. Even with this progress, key challenges remain how to further improve the efficiency of probes across the entire BBB; how to ensure absolute targeting specificity in such a complex *in vivo* environment and eliminate interactions with non-target proteins; and how to evaluate long-term *in vivo* safety and Gd clearance kinetics. In the future, the development of a new generation of probes that can actively target the BBB, intelligently respond to the aggregation state or pathological microenvironment, and integrate multimodal imaging functions will be a key direction for promoting early and precise imaging diagnosis of AD.

#### 3.1.2. Mn(II)-Based Probes

Mn(II) is an endogenous metal element that has five unpaired electrons and thus higher relaxivity than Gd(III); it is considered a potential alternative. The design concept for Mn-based probes is the same as for Gd probes, i.e., chelation of Mn(II) with specific ligands to form stable complexes. Because Mn²⁺/Mn³⁺ is an important biological element that exhibits paramagnetism, it is a potentially safer alternative to Gd for MRI probe design. For Mn(II)-based probes, the main strategy is to use the redox properties inherent in Mn or its integration into a biocompatible molecular structure to achieve targeted Aβ imaging. Initially, the researchers considered the direct use of manganese chloride as an MRI contrast agent. The Kim group demonstrated that MnCl_2_ could be used as MRI contrast by a two-mechanism approach 36. Mn^2+^ and Ca^2+^ can enter the brain through the calcium channels of neurons. This is because they are both divalent cations. Moreover, Mn ions can bind to the carboxyl group and the amino group in the Aβ aggregates, which is the basis for targeting. Once injected under the skin, this method causes a progressive accumulation of the metal in the brain. However, it does not specifically target the Aβ aggregates. In addition to the simple manganese salt, Martinho and Almeida Pinto’s team developed a versatile Mn(III) porphyrin complex 61. The project uses tetrapyrrole macrocycle as the main chelating ligand, and it is planned to add trifluoromethyl and PEG groups to develop an asthonic redox molecular probe. At a frequency of 20 MHz, the relaxation rate of the probe varies from 4.0 mM⁻¹ s⁻¹ for the oxidation state of Mn(III) to 6.8 mM⁻¹ s⁻¹ for Mn(II) in situ, which makes it possible to detect the in situ oxidation environment of tissues. Hydrophobic porphyrin rings are likely to interact with the β-sheet structure of Aβ. With Aβ_1-40_, the relaxivity of the porphyrins increases. This work represents obvious conceptual progress, but in practice it still faces problems related to delivery efficiency and *in vivo* stability. The synthesis of well-targeted and well-defined small Mn(II) molecular complexes represents another unique and rational design approach. Sharma et al. (2017) 35 created a series of Mn(II) complexes based on pentadentate ligands. The key innovation in their design is the incorporation of tryptamine (bearing an indole group) into one ligand (L₃) to serve as an Aβ targeting unit, while using structurally similar ligands without the indole (L₁, L₂) as negative controls. This design proves that the indole group mediates specific hydrophobic interactions with Aβ₄₂ fibrils, with MnL_3_ exhibiting a dissociation constant (Kd) of 3.19 μM. They also demonstrated dual T1/T2 modality, with r1 relaxivities (6.44 - 7.26 mM⁻¹ s⁻¹) exceeding that of clinical Gd-DTPA and high r2 values (23.26 - 37.86 mM⁻¹ s⁻¹), attributed to their rigid, heptacoordinate structure. This work provides clear guidance for constructing targeted, multimodal Mn-based probes, but as seen in all the above works, it has common problems of modest (μM) binding affinity compared to high-affinity Gd probes, and a lack of *in vivo* BBB penetration and imaging data.

Because of their endogenous metal properties, better biological metabolic pathways, and higher relaxation rate, Mn-based probes have shown potential application value; however, related research lags far behind that of Gd-based probes. Core bottlenecks are concentrated in several aspects: (1) Excessive accumulation of Mn²⁺ can easily cause manganese poisoning (such as Parkinson's symptoms), necessitating an ultra-stable chelation system and increasing synthesis difficulty; (2) The r₁ of the Mn complex is greatly affected by the coordination environment, and the increase in relaxation rate after binding to Aβ is lower than that of Gd probes; (3) Mn ions tend to bind non-specifically to other metal-binding proteins (e.g., transferrin) *in vivo*, interfering with imaging signals; (4) Most existing studies remain *in vitro* or short-term *in vivo*, lacking long-term biosafety verification, which limits their development scale. Therefore, current research focuses primarily on the development of high-affinity chelator systems to prevent *in vivo* dissociation of Mn²⁺ and on optimization of dosage regimens to reduce risks of neurotoxicity.

#### 3.1.3. ¹⁹F MRI Molecular Probes for AD Imaging

¹⁹F MRI is a novel molecular imaging technology that offers unique advantages for non-invasive and highly specific imaging of Alzheimer’s disease Aβ pathology, due to the few endogenous fluoride background signals present primarily in biological systems. There are almost none 67. The core of ¹⁹F-labeled probes technology lies in the rational design and application of ¹⁹F-labeled probes. To ensure the proper operation of ¹⁹F MRI, these probes must simultaneously meet a number of demanding requirements. The ¹⁹F probes can effectively cross the blood-brain barrier and reach effective concentrations in the brain parenchyma, with high affinity and the ability to selectively bind Aβ assemblies (including oligomers and fibrils), as well as maintain targeted binding in a complex hydrophobic brain microenvironment after binding, particularly by maintaining the T₂ relaxation properties, avoiding significant loss of signal. The development of ¹⁹F MRI probes is essentially an iterative innovation process focused on the progressive resolution of physico-chemical and biological challenges.

**Figure 6:**
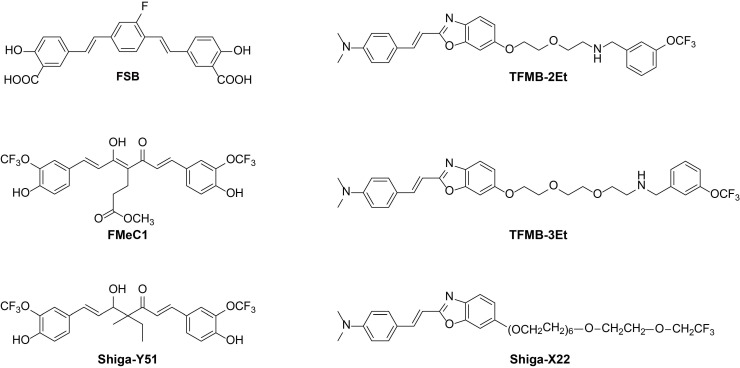
Chemical structures of ^19^F MRI probe for Aβ MR imaging. These examples were obtained from the following references: FBS^62^, FMeC1 64, TFMB-2Et and TFMB-3Et^63^, Shiga-Y51^39^ and Shiga-X22 65.

**Figure 7:**
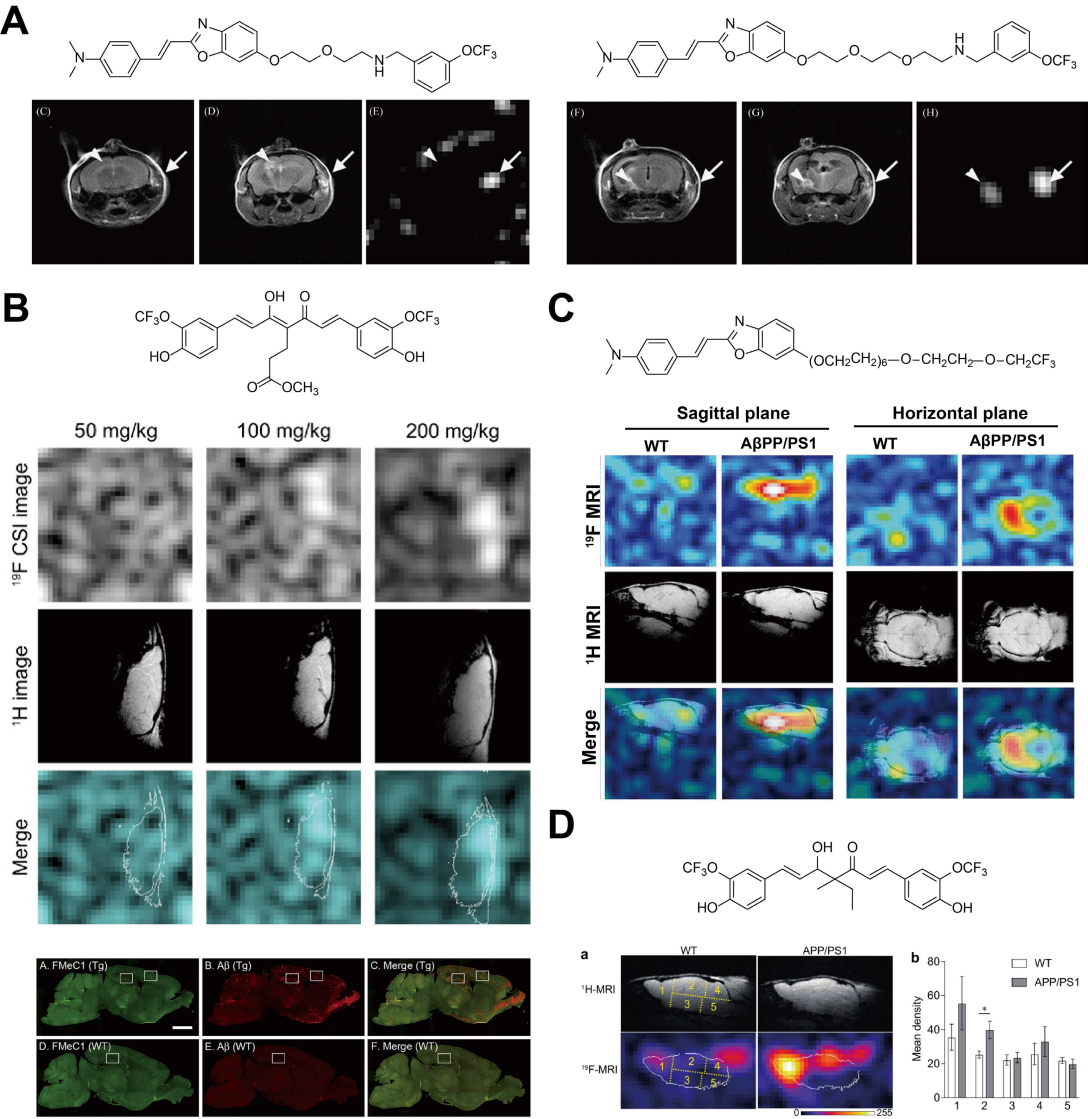
**^19^F probes used for MRI of Aβ species.** (A) Chemical structures of the fluorinated probes TFMB-2Et and TFMB-3Et, and their corresponding *in vivo* ¹H gradient echo images and co-registered ^19^F chemical shift images (CSI) in a representative animal model. Adapted with permission from 63, Copyright 2009, Elsevier. (B) ^1^H-MR images and ^19^F-MR images in WT and APP/PS1 mice that were obtained for 50 min, as well as the levels of ^19^F signals were measured in five regions indicated in yellow color. Adapted with permission from 64, Copyright 2011, Elsevier. (C) ^19^F MR signals in WT and APPswe/PS1dE9 doubletransgenic (APP/PS1) mice after injected with probes. Adapted with permission from 65, Copyright 2014, SAGE. (D) Dose-dependent ¹⁹F signal intensity measured by ^19^F NMR spectroscopy and ^19^F CSI in the brains of Tg2576 transgenic mice following FMeC1 injection, with corresponding ex vivo histological analysis of brain sections from the same model. Adapted with permission from 39, Copyright 2021, MDPI.

Early probe designs directly borrowed the scaffold of Aβ ligands validated in PET or fluorescence imaging and incorporated ¹⁹F atoms. The purpose of these works was to check feasibility and investigate basic structure-activity relationships (**Figure 6**). The pioneering work of Amatsubo et al. involved the synthesis of trifluoromethoxy benzoxazole derivatives TFMB 2Et and TFMB 3Et 63. They were the first to clearly observe that although both probes give sharp ¹⁹F NMR signals *in vitro*, the signals in brain tissue homogenates differ vastly: the signal of the more hydrophobic TFMB 2Et (log P = 6.40) is almost completely suppressed, while the slightly more hydrophilic TFMB 3Et (log P = 6.07) can still be partially detected. This directly shows that the highly hydrophobic myelin lipid environment in the brain is one of the “traps” leading to ¹⁹F signal attenuation, and enhancing probe hydrophilicity is the first guiding principle for early design. Research on ¹⁹F probes based on another classic Aβ ligand scaffold, distyrylbenzene, also supports this view (**Figure 7A**). Nabuurs et al. systematically synthesized 15 polyfluorinated derivatives. Some probes (e.g., compounds 13 and 22) have nanomolar level high affinity and excellent staining specificity for Aβ plaques in mouse and human AD brain sections and can cross the BBB 68. However, their very high hydrophobicity (log P generally >7) makes them unable to generate effective ¹⁹F MRI signals in the brain parenchyma. Earlier research by Flaherty et al. reported a similar dilemma: even though some polyfluorinated distyrylbenzene probes (e.g., compound 8) have ultra-high *in vitro* affinity for Aβ₁₋₄₀ fibrils at the picomolar level, their strong hydrophobicity leads to failure *in vivo* 38. These studies show that the probe requires a certain amount of lipophilicity (often associated with log P values) to prevent the problem of a lipid trap in the brain. Adequate hydrophilicity also requires an important design trade-off. These compromises aim to achieve the ideal log P in the 3-4 range. At the same time, researchers are investigating ways to improve the physicochemical properties of the probes, using chemical and kinetic properties of the targeting molecules to improve target binding and imaging effects. A typical example is the dual conformation probe developed by Yanagisawa and others called FMeC1. The probe uses the peculiar keto-enol tautomerism of curcumin's inherent backbone. In physiological conditions, it is mainly present in the enol form, but converts to the keto form upon interaction with Aβ. Since the keto form's structure interacts with the Aβ β-sheet structure, binding is enhanced by increased hydrogen bonding. These structural changes have not only strengthened the affinity with the target, but also prolonged the probe's residence time at the target. A study of Tg2576 transgenic mice has shown that the probe crosses the BBB and shows signal in cortical fibers and hippocampus and binds to Aβ. This study confirmed for the first time that environmentally responsive ¹⁹F probes have been developed, and provided an important model for further investigation 64 (**Figure 7B**).

Optimizing the hydrophilic-lipophilic balance solved the basic problems of a probe “reaching” and “surviving” at the target site. A more subtle problem is that when a probe molecule binds to rigid, highly ordered Aβ aggregates (especially fibrils), its overall molecular motion becomes severely restricted. Consequently, the local motional frequency of ¹⁹F nuclei decreases, leading to a sharp increase in the transverse relaxation rate (1/T2, r₂), i.e., significant T2 shortening and signal broadening. Even with sufficient brain concentration and appropriate hydrophilicity, the imaging signal remains weak. Such a “post-binding signal attenuation” problem was subsequently solved by the Yanagisawa team through a revolutionary “structural isolation” design strategy 65, and their representative probe Shiga X22 became a milestone in the field. The structural ingenuity of Shiga X22 lies in embedding a flexible PEG chain composed of 7 ethylene glycol units between the benzoxazole targeting core and the trifluoroethoxy (-OCF₂CF₃) signal unit. With this design, the functional modules are “decoupled”: the PEG chain acts as a long, flexible spatial spacer arm. Even after the targeting core becomes rigidified upon tight binding to Aβ fibrils, it effectively isolates and maintains a high degree of motional freedom for the ¹⁹F atoms in the terminal trifluoroethoxy group. Therefore, probe binding no longer inevitably leads to catastrophic ¹⁹F signal attenuation. With this design, Shiga X22 achieved, for the first time, specific and long duration (signal lasting up to 8 hours post injection) ¹⁹F MRI imaging of Aβ plaques in APP/PS1 transgenic AD mice (**Figure 7C**).

Once stable imaging of fibrillar plaques was achieved, the research frontier naturally moved toward the more neurotoxic early pathological species in AD pathogenesis—oAβ. This required probes not only to bind but also to differentiate between aggregation states. Yanagisawa et al. developed the probe Shiga Y51 through clever conformational engineering of curcumin, a natural Aβ binding molecule 39. By introducing methyl and ethyl substitutions at the C4 position, they “lock” the curcumin core into the more flexible ketone form instead of the enol form, which tends to form a rigid planar structure. This flexible ketone structure can more effectively embed into the loosely structured, dynamically changing hydrophobic core of oAβ but has difficulty binding to the tightly packed, structurally rigid mature Aβ fibrils. This probe achieved, for the first time, specific ¹⁹F MRI visualization of oAβ in the brains of AD model mice, with signal intensity positively correlating with ELISA-quantified oAβ levels (**Figure 7D**). The fluorinated curcumin probe Curc-Glu-F9, recently reported by Micocci and co-workers, has further validated and refined this strategy of morphological selectivity 66. When incubated with oligomeric Aβ, this probe retained a sharp, narrow ¹⁹F NMR peak; however, its signal was almost completely suppressed upon binding to mature amyloid fibrils.

In addition to achieving morphological selectivity, newly designed probes are also pursuing comprehensive performance improvements. Dai et al. developed probe 7d using the indanone framework derived from donepezil, a clinically used drug for Alzheimer’s disease 19. This probe showed balanced nanomolar affinity toward both Aβ₁₋₄₀ and Aβ₁₋₄₂, with dissociation constants around 370-380 nM. The log P value of the compound is 3.87, indicating its ability to pass the blood-brain barrier. In addition, its pharmacokinetic properties in the body are favorable: brain uptake reached a maximum within 1 hour, and the compound was almost cleared in 24 hours. The compound has relatively low acute toxicity and its LD₅₀ values exceed 50 mg/kg, indicating good potential for future clinical use. In essence, the field of ¹⁹F MRI for Aβ imaging has broken through the first challenges regarding hydrophilicity and signal disruption. It has reached a stage where it is possible to distinguish between certain forms of Aβ aggregation, especially oligomers and protofibrils. The current core design strategy focuses on fine-tuning the molecular hydrophilic-lipophilic balance to maintain BBB permeability and stable imaging signals, and using flexible linkers or conformation-dependent interactions to maintain signal strength and target selectivity after binding. However, this technology faces a number of important limitations. Most probes usually require a relatively high dose on the order of 200 mg/kg, which means that long-term biosafety needs to be thoroughly assessed. Image sensitivity is also highly dependent on high magnetic field (≥ 7.0 T) MRI scanners. The future direction of development should focus on several areas: (1) developing probes with higher intrinsic sensitivity by increasing affinity to the target and lowering the effective dose by, for example, optimizing the number of ¹⁹F atoms and their spatial location; (2) further exploring probes that can absolutely distinguish between different Aβ aggregation forms; (3) performing validation studies on multimodal imaging platforms; and (4) working to build probe systems compatible with clinical MRI scanners and accelerate clinical translation.

#### 3.1.4. Structural Optimization Strategies from PET to MRI Probes

A common design method is converting a PET tracer to an MRI probe. This method utilizes a PET ligand-specific target framework while adjusting the molecular structure to meet the specific physicochemical requirements of MRI. The transformation is not just a direct replacement of radioactive nuclei. Instead, researchers need to make detailed structural optimizations to harmonize considerations of affinity, blood-brain barrier permeability, relaxivity (or ¹⁹F signal stability), and biosafety. **Table 2** summarizes typical cases and highlights important structural changes and their impact on imaging performance. MRI has three significant advantages over PET. First, it makes it possible to eliminate ionizing radiation completely and allow secure and repeated longitudinal monitoring (e.g., scanning quarterly) without worrying about accumulated radiation exposure, which is important for tracking disease progression before symptoms appear. Second, MRI has better spatial resolution (as low as ~100 μm in high-field animal MRI, compared to 3-5 mm for PET), which allows for precise anatomical localization of Aβ deposits in small brain areas like the hippocampus and cortex. Third, MRI is inherently suitable for theranostic platforms. The same targeting scaffold used to conjugate a contrast agent can also be linked to a therapeutic agent (such as anti-aggregation compounds or immunomodulators), which makes it possible to establish a closed-loop “diagnosis-therapy-evaluation” model, something that cannot be done with PET.

### 3.2. Nanoparticle Based Probes

Nanoprobes use nanomaterials (e.g., SPIONs, liposomes, mesoporous silica) as carriers and are conjugated with targeting moieties via surface modification. Nanoprobes are supramolecular assemblies with sizes of 1-100 nm. Owing to size effects and surface functionalization ability, they provide novel solutions for Aβ imaging. Compared with small molecule probes, nanoparticles have higher loading capacity and greater potential for multifunctional integration, i.e., multimodal imaging modalities (**Table 3**).

#### 3.2.1. T2 Weighted MRI for AD Diagnosis

SPIONs, as high performance T2/T2* weighted MRI contrast agents, have the advantage of non-invasive visualization of Aβ pathology in AD by shortening the transverse relaxation time of surrounding water protons. The core developmental track of this field is how to engineer SPIONs, through sophisticated surface modification, to cross the intact BBB with high relaxivity and achieve specific targeting and enrichment of Aβ aggregates. Research in this field is moving from proof-of-concept studies to a stage of functional integration and intelligent theranostic design.

**Figure 8:**
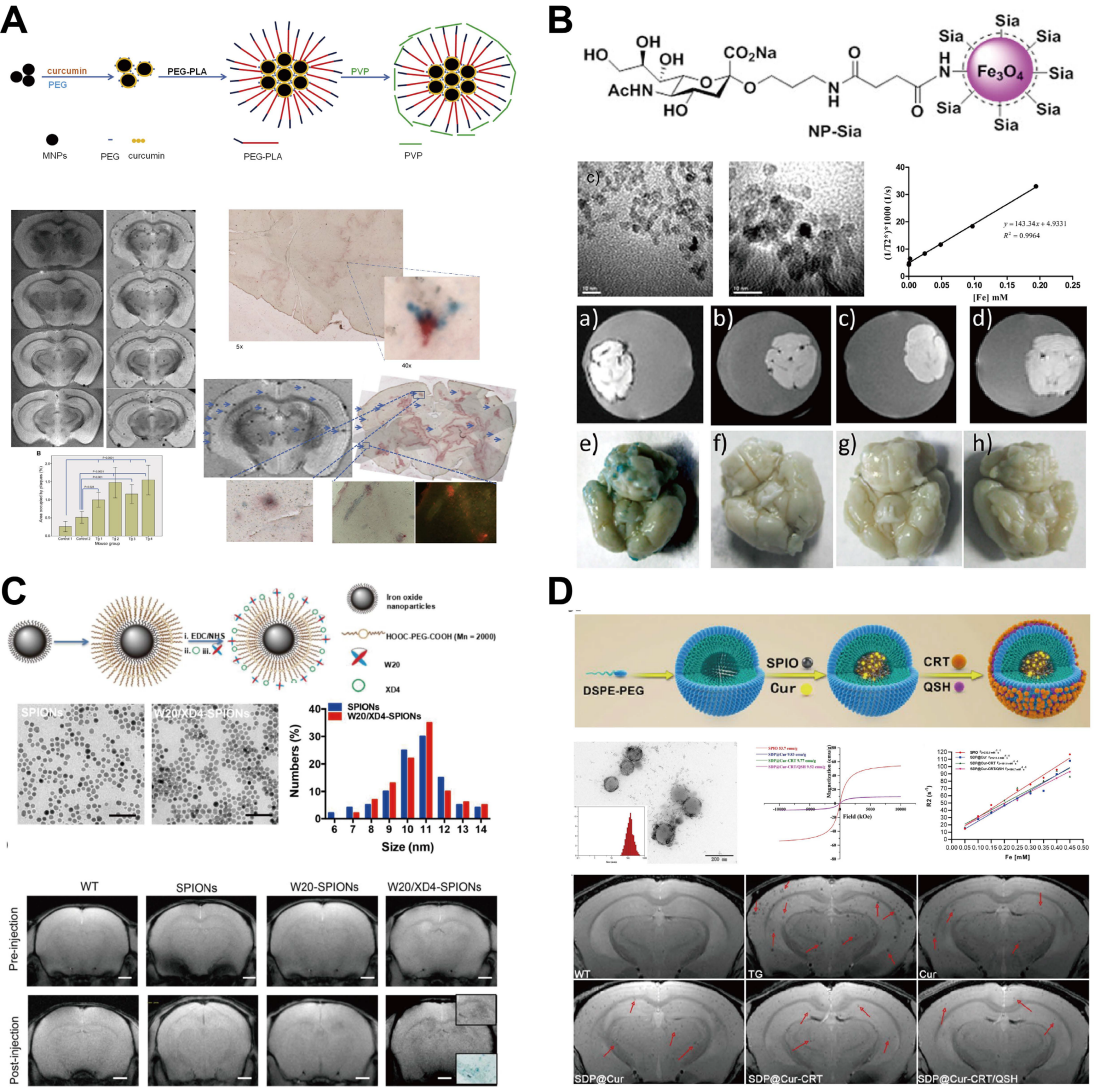
** T2-weighted MR nanoprobes for Aβ species.** (A) Synthesis scheme of Cur-MNPs, ex vivo T2*-weighted MR images, and corresponding histochemical staining of brain sections from 18-month-old Tg2576 transgenic mice and age-matched wild-type controls following systemic injection of Cur-MNPs. Adapted with permission from 37, Copyright 2015, Elsevier. (B) Structure, TEM image, T2* relaxivity, and T2*-weighted MR images of NP-Sia under different conditions after incubation with Aβ-containing mouse brain homogenates or brain sections. Adapted with permission from 82, Copyright 2013, American Chemical Society. (C) Preparation and characterization of W20/XD4-SPIONs, and *in vivo* T2*-weighted MR images of AD mouse brains at selected time points after intravenous injection. Adapted with permission from 72, Copyright 2020, Dovepress. (D) Synthesis and characterization of the SDP@Cur-CRT/QSH nanocomposite, and coronal T2*-weighted MR images of WT and transgenic (TG) mice treated with either PBS (control) or Cur-MNPs for three months. Adapted with permission from 85, Copyright 2022, Springer Nature.

Early explorations mainly focused on validating the targeting efficacy of conjugating classic small molecule Aβ ligands to SPIONs. For example, Zhou et al. and Zhang et al. used a ligand exchange strategy to modify the SPION surface with high affinity DDNP carboxyl derivatives and confirmed their specific binding to Aβ fibrils and their potential as T2 contrast agents 69,70. Cheng et al. used non-covalent interactions to load the natural multi-functional molecule curcumin onto SPIONs stabilized with PEG PLA/PVP. This probe achieved specific *in vivo* MRI imaging of Aβ plaques in the cortex and hippocampus of Tg2576 transgenic AD mice and showed good biocompatibility and potential for BBB penetration 37 (**Figure 8A**). To enhance relaxivity, Zeng et al. chose the clinically validated PiB to modify Mn-Zn ferrite nanoparticles and achieved an excellent r₂ relaxivity as high as 169.93 mM⁻¹ s⁻¹, much higher than traditional iron oxide contrast agents 71. Such probes, which rely mainly on passive diffusion, often suffer from insufficient delivery efficiency to support high sensitivity imaging and lack selectivity for different Aβ aggregation states.

To break the BBB barrier and achieve active targeting, research strategies turned to precision designs guided by biomacromolecules. Antibody-mediated targeting offers extremely high specificity. Sillerud et al. covalently conjugated an anti-APP antibody to SPIONs and found that the number of Aβ plaques detected in APP/PS1 transgenic mice nearly doubled without the help of BBB opening agents 73. Uranova and her team developed a nanoprobe with an iron core and an iron oxide outer shell, where DMSA was used to modify anti-Aβ antibodies. They used proteomics technology to systematically assess this probe's excellent biocompatibility and provided a new model to assess the probe's safety. Yan et al. used a "homogeneous measurement" method to conjugate the entire length of Aβ_1-42_ peptides onto a very small SPION. This design makes it possible to target Aβ deposits in the brain through hydrophobic interactions between peptides. Usually, this method needs co-administration of mannitol to temporarily open the BBB, which adds an extra risk for clinical translation 80.

Since the molecular size of full antibodies may limit penetration efficiency, smaller targeting modules such as cell-penetrating peptides and specific peptide sequences were favored. Xiong et al. co-conjugated the HIV-1 Tat protein transduction domain (Tat PTD) and the Aβ core recognition fragment Aβ (16-20) to ultrasmall SPIONs 81. Taking advantage of the potent membrane translocation capability of Tat PTD, this probe promoted BBB penetration and achieved precise imaging of Aβ plaques in APP/PS1 mice. Targeting strategies based on natural biomolecules have also attracted attention. For example, sialic acid was used as a targeting ligand, which can bind to histidine residues of Aβ via hydrogen bonding (**Figure 8B**), as explored by Kouyoumdjian et al. and Nasr et al 82. The cationic BSA-sialic acid-SPIONs probe designed by Nasr et al. even achieved specific imaging of Aβ plaques in APP/PS1 mice *in vivo* without the aid of mannitol 83. Some studies explored non-traditional targets. For example, Mundt et al. attempted to target activated microglia via intracerebroventricular injection of ultrasmall SPIONs to indirectly reflect peri-plaque inflammation, but the probes failed to diffuse effectively into plaque regions 84.

The most advanced progress in this field is demonstrated by the paradigm shift from “imaging only” to “theranostics.” Liu et al. designed a dual-function probe equipped with a single-chain variable fragment antibody (scFv W20) that recognizes oAβ and a heptapeptide (XD4) that can activate the class A scavenger receptor on microglia 52. It not only enabled MRI detection of oAβ, a key pathological species in early AD, but also promoted its phagocytic clearance by microglia, representing a preliminary synergy between diagnostic and therapeutic potential for AD 72 (**Figure 8C**). The work by Ruan et al. built a more complex multifunctional nanoplatform integrating SPIONs (imaging unit), curcumin (anti-inflammatory therapeutic unit), a transferrin receptor targeting peptide CRT (BBB penetration unit), and an Aβ targeting D peptide QSH. In the APP/PS1 mouse model, the platform achieved 3D MRI-based quantitative monitoring of Aβ plaque burden and simultaneously exerted neuroprotective and cognitive improving effects through sustained release of curcumin, representing the current pinnacle of theranostic design 85 (**Figure 8D**). Additionally, Lai et al. reported a very innovative “in situ biosynthesis” strategy. They simply intravenously injected AD mice with zinc and iron precursor compounds and exploited the unique high reactive oxygen species microenvironment within the AD brain to induce in situ formation of ZnO/Fe₃O₄ nanoclusters at Aβ deposition sites, thereby enabling fluorescence/MRI dual-modal imaging. Although this strategy circumvented complex *in vitro* synthesis and BBB penetration problems, the specificity and intensity of its imaging signal are heavily dependent on the pathological microenvironment, challenging the universality and stability of this strategy 78.

There are remarkable technical advances in the application of SPIONs for Aβ imaging, which have evolved from simple ligand modifications to active targeting strategies and even further to integrated multifunctional smart probes. To date, the most advanced probe is a theranostic probe that integrates high-sensitivity MRI imaging, effective active BBB delivery, and potential neuroprotective functions. With advances in nanoparticle design, particularly in targeting precision, BBB penetration capability, and the increase of multiple functions, targeted SPIONs have become a powerful and adaptable tool for non-invasive early diagnosis and therapeutic monitoring of Alzheimer’s disease (AD). Future research should focus on the development of multimodal probes. These probes combine magnetic resonance imaging (MRI) with other imaging methods. On the other hand, it is also necessary to improve smart probes to identify specific Aβ forms. In addition, it is extremely important to optimize the dosing schedule for future clinical applications. This new technology could radically change the understanding and management of Alzheimer’s disease. It allows for early intervention that can fundamentally change the course of the disease.

#### 3.2.2. T1 Nanoprobes

The development of T1-weighted nano-sized MRI probes represents an important advance in the non-invasive detection of β-amyloid pathology in the brain in Alzheimer's disease. It is difficult to distinguish signals from T2* contrast from low signal artifacts such as bleeding and calcification. In contrast, T1-weighted probes generate a positive contrast, making it easier to interpret the results and perform anatomical co-localization. Recent years of research have primarily focused on the manufacture of multifunctional nanoparticles based on Gd or Mn. The researchers also seek to improve target specificity and image sensitivity by systematically optimizing the size, surface chemical properties, and functional ization methods of these nanoparticles. Previous studies focused on examining whether binding of Aβ-targeted ligands and paramagnetic ions (such as Gd) is possible. Li et al. combined curcumin derivative (CR) with bovine serum albumin-Gd chelate to create the probe precursor CR-BSA-(Gd-DTPA). This study showed that this probe can bind specifically to Aβ fibrils *in vitro* and induce changes in the T1 relaxation rate, which has formed the basis for the development of small molecule targeted probes. However, these initial trials had obvious drawbacks, being difficult to cross the BBB effectively and unable to distinguish between different Aβ structures. Therefore, the researchers shifted attention to an active targeting strategy that increases target specificity. This is represented primarily by antibodies, peptides or small-molecule ligands. Antibodies are known for their high binding affinity. For example, He et al. conjugated Aβ antibodies (4G8) to their probes and targeted soluble and insoluble Aβ species. Jaruszewski et al. modified the nanocarrier using IgG4.1 antibodies and targeted cerebrovascular amyloid (CVA), while MRI/SPECT imaging can be combined with the delivery of anti-inflammatory drugs.

**Figure 9:**
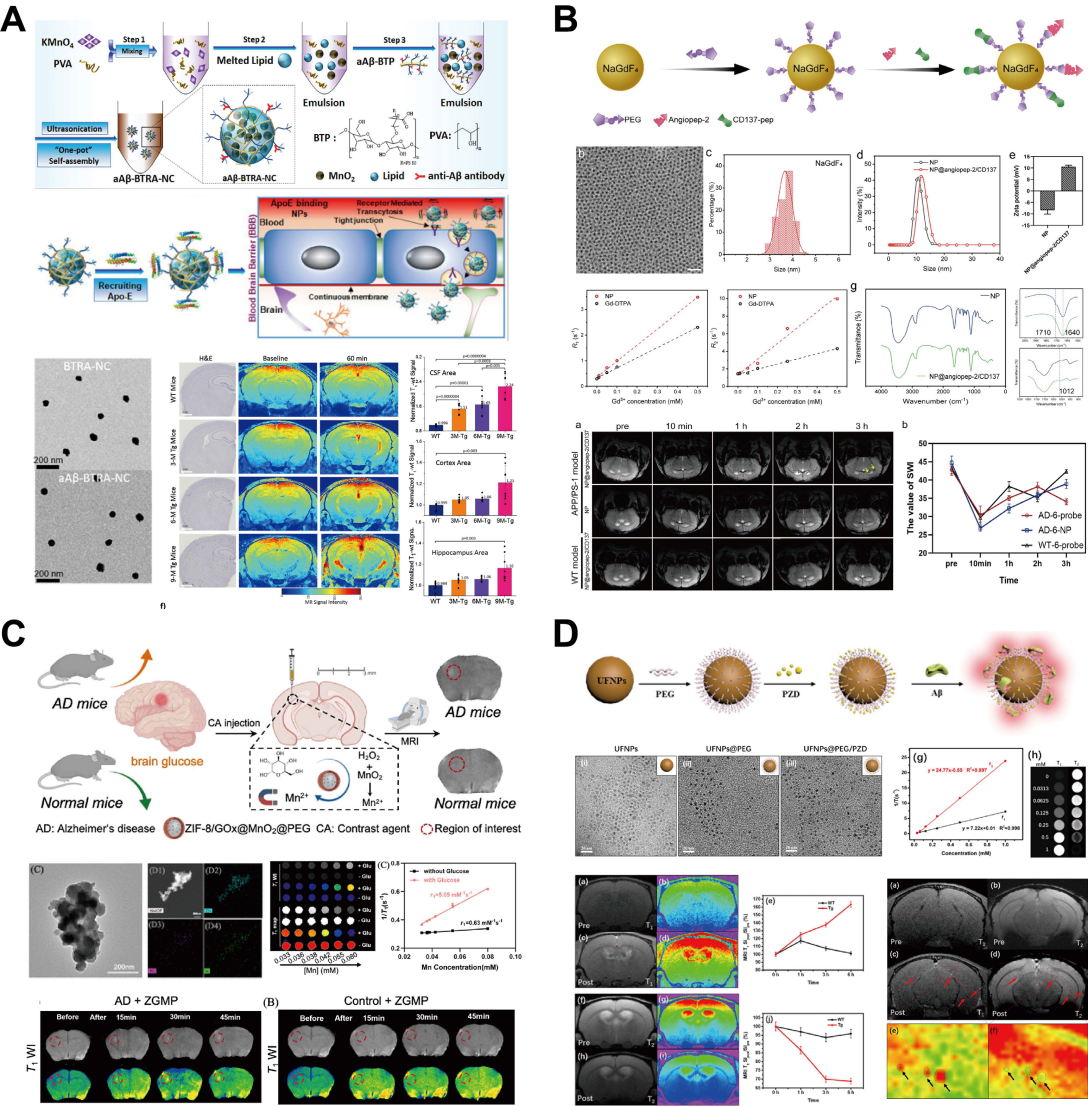
** T1-weighted nanoprobes for Aβ species MR imaging.** (A) *In vivo* axial T1-weighted MR images of WT and Tg mice at different ages, acquired before (baseline) and 60 min after treatment with aAβ-BTRA-NC. Adapted with permission from 86, Copyright 2020, Elsevier. (B) Synthesis scheme, physicochemical characterization, and *in vivo* T2/T2*-weighted MR images of APP/PS-1 transgenic and WT mice at different time points after intravenous injection of NP@angiopep-2/CD137. Adapted with permission from 74, Copyright 2025, Royal Society of Chemistry. (C) Brain glucose activated MRI contrast agent for early diagnosis of AD characterization of ZGMP. Adapted with permission from 90, Copyright 2022, American Chemical Society. (D) Fabrication, *in vitro* MR signal response to Aβ aggregates, and *in vivo*T1/T2-weighted MR images of a transgenic mouse before and after intravenous injection of UFNPs@PEG/PZD. Adapted with permission from 76, Copyright 2020, American Chemical Society.

To address the limitations of antibodies, namely their large molecular size and high cost, researchers developed smaller targeting modules 87. Plissonneau et al. used short Aβ-derived peptides to modify ultrasmall Gd-based nanoparticles to achieve specific recognition of Aβ fibrils and demonstrated potential for typing diagnostics 88. Tanifum et al. synthesized a hydrophilic small molecule ligand, ET6-21, for liposome modification; with this “dual Gd” design, they achieved high relaxivity and successful *in vivo* imaging at low field (1T) MRI 77. Efficient BBB penetration is a must for probe efficacy. Besides passive diffusion, active receptor-mediated transcytosis strategies have been widely adopted. The probe by He et al. used a brain-targeting polymer to adsorb ApoE to mediate LDLR-dependent transport 86 (**Figure 9A**); the probe by Zhou et al. used the RVG29 peptide targeting nAChR to improve brain delivery efficiency of their PLA-PEG nanoparticles loaded with rifampicin and Gd, for both therapeutic effect and treatment monitoring 89; the probe by Jiang et al. extended the target to neuroinflammation. Their probe combines the BBB-penetrating peptide angiopep-2 with a CD137 antibody to visualize inflammatory foci in early AD 74 (**Figure 9B**).

To obtain higher imaging specificity, environmentally responsive “smart” probes have been developed. Such probes are designed to be turned on in situ; that is, the signal turns on when the probe encounters specific pathological microenvironmental triggers such as high ROS levels or abnormal glucose concentration. The seminal work by He et al. used MnO₂ nanoclusters as the core; in the presence of H₂O₂, Mn²⁺ ions are released. This not only increases the r1 value but also consumes pathological ROS, representing true theranostic integration 86. Liu et al. utilized the abnormal glucose metabolism often present in early AD to construct a GOx-loaded ZIF-8@MnO_2_ probe, enabling glucose concentration-dependent signal activation 90 (**Figure 9C**).

Current frontiers are in theranostic integration and multimodal strategies. Besides the aforementioned exemplary studies, other multimodal probes provide more complete information by fusing complementary imaging modalities. Notably, Cai and his team reported a T1-T2 dual-mode probe based on ultrasmall ferroxymini nanoparticles. This probe targets Aβ aggregates by surface modification of phenothiazine derivatives (PZD). The extremely small size (less than 15 nm) significantly increases the efficiency of passive BBB penetration (**Figure 9D**). The property of this probe is that it simultaneously has a marked effect on both T1 and T2 relaxation times, and thus can provide synchronized “bright” T1 positive contrasts and “dark” T2 negative contrasts during visualization. This complementary mechanism of double signals makes it possible to effectively avoid false positivity or false negativity due to magnetic field inhomogeneity or magnetization rate artifacts. This will significantly improve the positional accuracy and spatial resolution of *in vivo* Aβ plaques, representing an important development direction for future highly reliable diagnostic probes.

Although these advances are very promising, there are still challenges in the clinical application of T1 nanoprobes. First, it is crucial to achieve optimal pharmacokinetic properties. This means that the probe must maintain sufficient concentration in the brain and minimize systemic exposure to the rest of the body. There is also a need for a comprehensive assessment of long-term biosafety, with particular attention to the accumulation of metals in neural tissues. In addition, the development of scalable production processes is crucial to ensure quality consistency between different batches. Future research should focus on three main areas: the development of next-generation probes with stronger binding specificity for various Aβ species (particularly soluble oligomers), the construction of intelligent stimuli-response systems that are activated only in the presence of specific disease markers, and the development of engineering integration platforms that integrate precision imaging and multiple therapies. Such efforts will pave the way for early and accurate diagnosis and intervention of Alzheimer’s disease, even at stages that are currently considered impossible to treat.

### 3.3. Multimodal Probes

The pathological development of Alzheimer's disease (AD) is associated with abnormal accumulation of Aβ plaques. Therefore, early, non-invasive and accurate imaging of Aβ deposits is extremely important, which is crucial not only for the diagnosis of AD, but also for discovering the underlying mechanisms of the disease. Each imaging modality has its own limitations. For example, MRI has a high spatial resolution and deep tissue permeability, but its sensitivity at the molecular level is relatively low. On the other hand, optical imaging techniques such as near-infrared fluorescence show high molecular sensitivity, but there are limitations due to low tissue penetration and autofluorescence interference from the tissue itself. To overcome these bottlenecks, multimodal imaging probes have been developed that combine MRI with other imaging modalities (fluorescence, X-ray phase contrast or ¹⁹F MRI). These probes integrate the advantages of various imaging methods and overcome the limitations of single-modal images by leveraging the high spatial resolution of MRI, the high sensitivity of optical imaging, and the quantitative accuracy of ¹⁹F detection. The design of a typical multimodal probe is based on functional nanomaterials or molecular couplings and comprises the following three main components: a targeting unit to identify Aβ deposits, an MRI contrast unit (typically paramagnetic Gd³⁺, superparamagnetic iron oxide or ¹⁹F labels), and an imaging unit (fluorescent or X-ray phase contrast) that acts as a molecular tracer. These multimodal probes allow for precise positional and quantitative measurement of Aβ deposits, as well as real-time monitoring of the course of the disease, and they form the basis for clinical diagnosis and therapeutic intervention of AD.

**Figure 10:**
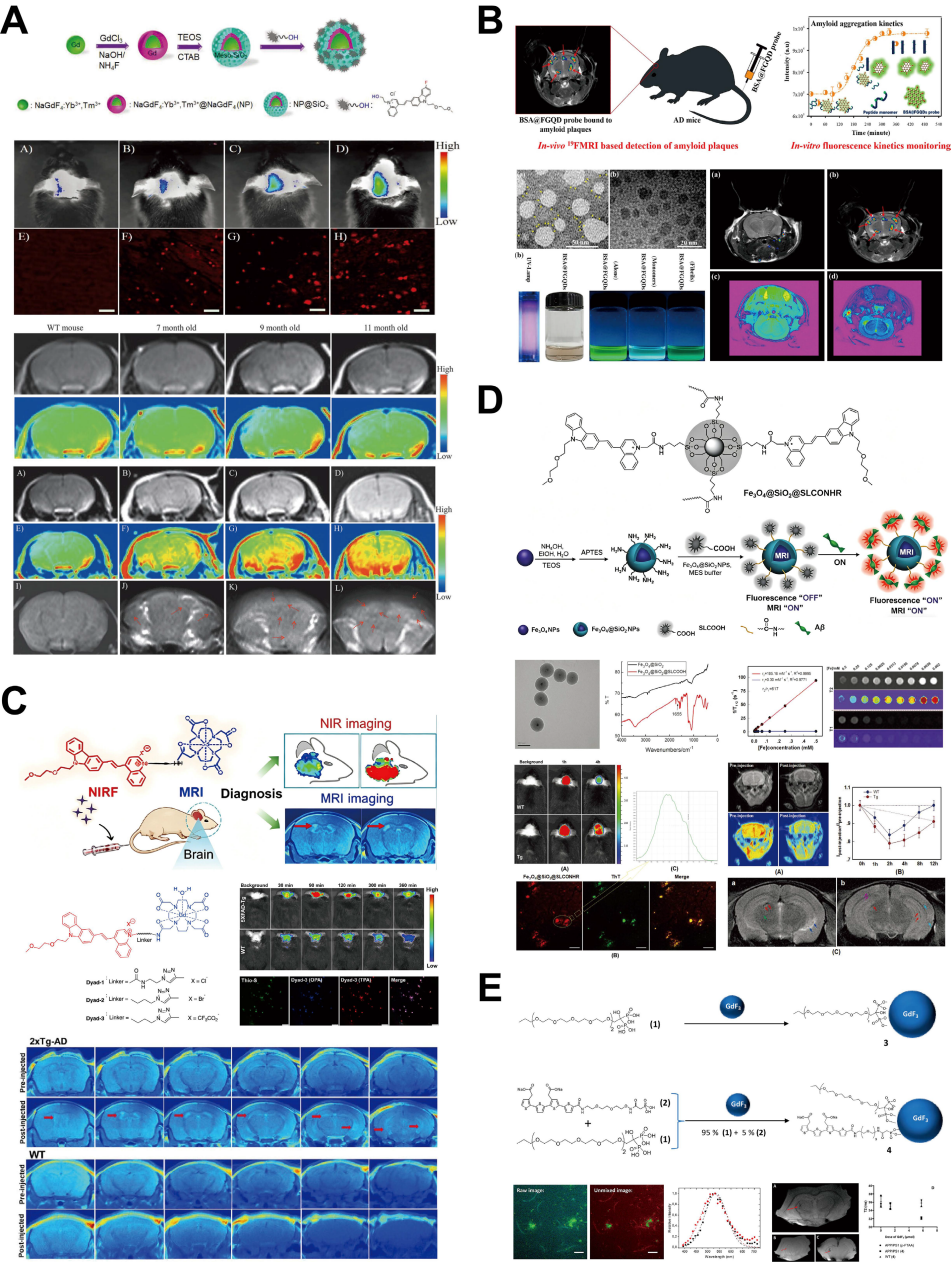
** Multimodal MRI probes for Aβ species imaging.** (A) Synthesis scheme of NP@SiO_2_@F-SLOH, ex vivo fluorescence staining of brain slices, and *in vivo* MR images of WT and Tg mice at different ages before probe injection. Adapted with permission from 92, Copyright 2020, Wiley-VCH. (B) Morphology and physicochemical properties of the BSA@FGQDs probe, and corresponding *in vivo* ^1^H MRI and ^19^F MRI signals of the mouse brain before and after its systemic injection. Adapted with permission from 95, Copyright 2019, American Chemical Society. (C) Molecular structures of the Gd(DOTA)-based dyad probes and *in vivo* dual-modal fluorescence/MR images of 12-month-old 5XFAD transgenic mice and age-matched WT mice before and after intravenous administration of Dyad-3. Adapted with permission from 52, Copyright 2020, American Chemical Society. (D) Fabrication of Fe_3_O_4_@SiO_2_@SLCONHR, its *in vitro* fluorescence/T1-/T2-weighted MR signal response toward Aβ aggregates, and ex vivo T2-weighted MR images of Tg mouse brains 4 h post-injection. Adapted with permission from 98, Copyright 2018, Wiley-VCH. (E) Two-photon fluorescence and MR-specific imaging of Aβ amyloid using hybrid nano-GdF_3_ particles. Adapted with permission from 99, Copyright 2018, American Chemical Society.

Among these designs, Gd³⁺ based T1 weighted contrast agents are in the majority, and relaxivity is the core metric for probe performance. Recently, Yang et al. reported a dual-functional probe, Gd NP@SiO₂@HY5, with “Aβ targeting and H₂O₂ responsive” properties 91. The nanoparticle used as the MRI core is NaGdF₄, coated with mesoporous SiO₂ and loaded with the near-infrared fluorescent probe HY5. The innovation is its dual response: on one hand, HY5 binds to Aβ₄₂ fibrils via hydrophobic interactions, giving about 30-fold fluorescence enhancement; on the other hand, H₂O₂ generated in the Aβ plaque microenvironment can oxidize HY5 to HY4, causing a blue shift in the fluorescence emission peak from 660 nm to 500 nm, enabling ratiometric detection of AD pathology related oxidative stress levels. *In vivo* experiments showed that this probe could clearly distinguish brain signals between APP/PS1 transgenic mice of different ages and WT mice, with both MRI and fluorescence signal intensities positively correlated with the degree of Aβ deposition. Targeting the more neurotoxic oAβ, Wang et al. developed a theranostic probe, NP@SiO₂@F SLOH 92. They used a core-shell structure of NaGdF₄:Yb³⁺,Tm³⁺@NaGdF₄ to ensure high relaxivity and loaded the fluorescent dye F SLOH, which selectively recognizes oAβ, via mesoporous SiO₂. This probe not only enables *in vivo* monitoring of dynamic oAβ enrichment with age (e.g., inducing about 60% MRI signal enhancement in the brains of 11-month-old AD mice), but the loaded FSLOH also effectively inhibits Aβ fibrillization and reduces Aβ-induced cellular reactive oxygen species levels by about 40%, meaning “imaging and therapy” can be synchronized (**Figure 10A**).

Modular design and multifunctional integration are also trends for improving probe application flexibility and utility. Pansieri et al. used the AGuIX nanoplatform, well known for high relaxivity and ultra-small size, to construct modular bimodal probes by surface modifying with either the classic Aβ ligand PiB or the high-affinity nanobody B10AP 97. This design showed that one platform could be flexibly adapted to different targets and amyloid diseases (e.g., AD, type 2 diabetes); the affinity of the antibody-modified probe was several orders of magnitude higher than that of the small molecule ligand probe. In terms of molecular theranostic integration, Wang et al. synthesized the Gd(DOTA) cyanine dyad, Dyad 3 52. This small molecule probe covalently links an MRI contrast unit with a moiety having Aβ binding, aggregation inhibiting, and near-infrared fluorescence emitting functions. In AD model mice, it achieved near-infrared fluorescence imaging peaking at 90 minutes and clear MRI localization, and it also effectively inhibits Aβ fibrillization with a half-maximal inhibitory concentration in the μM range (**Figure 10C**). Probes based on superparamagnetic iron oxide, e.g., the USPIO NIR fluorescent probe developed by Li et al., take advantage of their T2* negative contrast effect and optical signal to provide a radiation-free bimodal imaging alternative 98 (**Figure 10D**). The GdF₃ two-photon fluorescence probe by Mpambani et al. uses two-photon excitation to achieve high-resolution imaging of Aβ plaques in deeper tissue 99 (**Figure 10E**).

Although multimodal MRI probes show great promise in preclinical research, translation to the clinic still faces a series of challenges. First, efficient BBB penetration is not universal. Most probes rely on the fact that the barrier function is impaired in the pathological state or on passive diffusion; thus, efficiency is very limited under physiological conditions. Second, long-term biosafety must be comprehensively evaluated. Metal-free probes offer an alternative path, but the potential central nervous system retention and neurotoxicity of Gd-based nanomaterials still need to be considered. Third, when translating from high-field preclinical MRI to widely available clinical equipment, contrast efficacy may decrease, posing a signal-to-noise ratio problem. Additionally, most existing probes target Aβ fibrillar plaques, while probes with high selectivity for the more toxic Aβ oligomers remain rather scarce, and attention to tau, another key pathological protein in AD, is also insufficient.

Through clever combination of various imaging mechanisms and functional units, multimodal MR probes have greatly pushed Aβ imaging technology toward earlier, more precise, more dynamic, and more functionalized development. From responsive probes and theranostic designs to modular, multi-target platforms, the above-mentioned strategies not only contribute to better understanding of the complex pathological mechanisms of AD but also provide rich and powerful toolboxes for future early diagnosis, efficacy monitoring, and even targeted therapy. **Table 4** summarizes typical examples of multimodal probes in Aβ imaging. Future research efforts should focus on improving probe BBB penetration efficiency, systematic verification of long-term biosafety, improved imaging performance under clinically relevant field strengths, and ultimately promoting these advanced imaging tools toward rigorous clinical trials and applications.

### 3.4. CEST MRI Probes for Aβ Imaging

CEST MRI is an emerging molecular imaging technique that generates contrast by detecting chemical exchange between exchangeable protons (e.g., from hydroxyl or amine groups) in solutes and bulk water protons. In the free state, exchangeable protons (e.g., hydroxyl and amide groups) on CEST probes undergo slow exchange with bulk water protons. The proton exchange rate (kₛw) is in dynamic equilibrium, and the CEST signal is correspondingly weak. Once the probe molecules bind to aggregated Aβ species, they undergo conformational changes, and the exchangeable protons interact with the hydrogen bond network on the surface of Aβ molecules. This increases kₛw by 3- to 5-fold and simultaneously prolongs T1, thus achieving targeted activation and specific amplification of the CEST signal.

Compared to T1-weighted imaging, which relies on paramagnetic metal ions (e.g., Gd³⁺, Mn²⁺), CEST probes do not require metal introduction, so they may have superior biosafety and a richer, more designable chemical space. They allow us to probe biological processes such as glucose metabolism, specific protein interactions, or enzyme activity, which is a unique advantage for imaging Aβ pathology in AD. Based on their origin and mechanism, current CEST probes for Aβ imaging mainly include endogenous metabolite-based probes, exogenous targeted peptide probes, enzyme responsive probes, and probes developed from repurposing of classic ligands. The relevant typical examples are summarized in **Table 4**.

**Figure 11:**
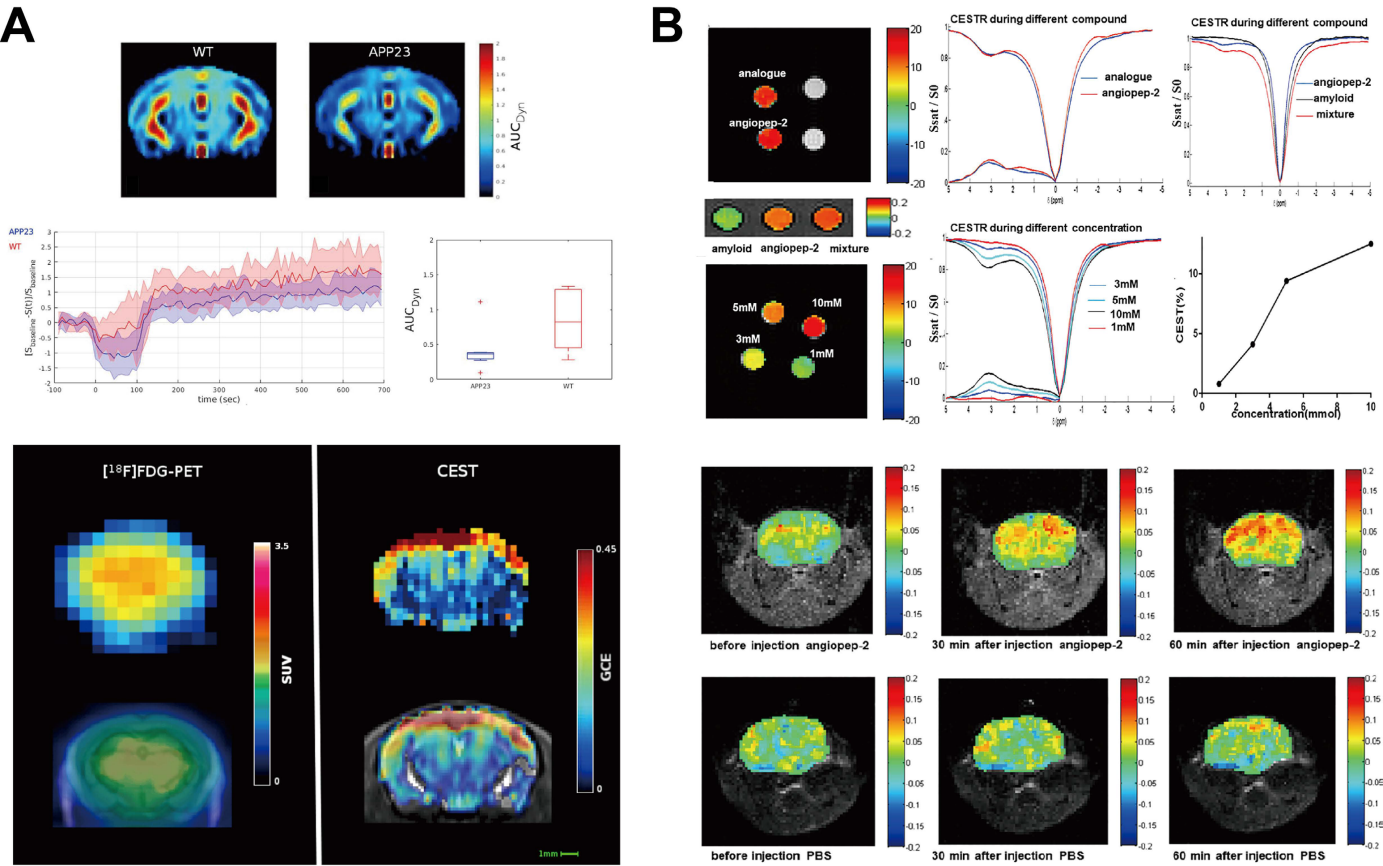
** CEST MRI probes for Aβ species imaging.** (A) Dynamic CEST measurements and comparative visualization of [¹⁸F]FDG-PET and CEST data. Top: Group mean AUC_Dyn_ images normalized to the template. Middle: Group mean dynamic signal curves (averaged from the cortex) and corresponding box plot of cortical AUC_Dyn_. Bottom: Representative [¹⁸F]FDG-PET and CEST images, alongside the multi-step fitting procedure: contributions of water and NOE were fitted first, followed by contributions from other compounds. Adapted with permission from 100, Copyright 2018, Springer Nature. (B) Z-spectra and CEST images of angiopep-2 and its analogue solutions *in vitro*, and longitudinal *in vivo* CEST images of APP/PS1 Tg mice (with PBS-treated mice as controls) acquired at different time points post-injection. Adapted with permission from 41, Copyright 2019, American Chemical Society.

Endogenous metabolite-based probes directly use intrinsic brain biomolecules or their analogs as reporting sources, introducing no exogenous substances. Tolomeo et al. were the first to show that the glucose analog 2-deoxy-D-glucose (2DG) can be an endogenous CEST probe 100; they demonstrated that the hydroxyl groups on the phosphorylated intracellular product, 2DG-6-phosphate (2DG6P), have exchangeable protons that can generate a CEST signal. In AD model mice, because cerebral glucose metabolism is diminished, uptake and retention of 2DG are reduced, and the CEST signal (GlucoCEST effect, GCE) is much lower than in WT mice. This study achieved the first non-radioactive imaging of brain glucose metabolism, providing an MRI based alternative to ¹⁸F-FDG PET (**Figure 11A**).

To obtain direct and specific imaging of Aβ deposition, various exogenous Aβ targeted peptide probes have been prepared. Wang et al. first reported an artificial peptide probe, Angiopep-2, possessing both BBB penetration and Aβ targeting capabilities 41. This probe crosses the BBB by binding to low-density lipoprotein receptor-related protein 1 (LRP1) and specifically binds to Aβ fibrils. Its amide/hydroxyl protons give a large CEST signal at 3.2 ppm. Injection of this probe into APP/PS1 mice produced remarkable enhancement of the CEST signal in the cortex and hippocampus, and the distribution was highly consistent with Aβ plaques revealed by immunohistochemistry, confirming its targeted imaging capability (**Figure 11B**). Xu et al. subsequently optimized imaging parameters for an Angiopep-2 based probe and systematically studied its use in dynamic monitoring of AD progression. They found that in APP/PS1 mice, the CEST signal intensity of this probe increases linearly with age from 6 to 12 months, and signal enhancement precedes the onset of cognitive impairment, allowing long-term, non-invasive monitoring of Aβ pathology from early deposition to progression 42.

Enzyme responsive CEST probes targeting AD related pathological processes, in addition to directly targeting Aβ protein, also have potential. Suchý et al. designed a dual-modal (CEST/fluorescence) probe targeting Cathepsin D (Cat D) 101. Cat D is overexpressed in the AD brain and can specifically cleave the linker peptide in this probe, releasing an active fragment containing thulium ions (Tm³⁺), which generates MRI contrast via the Off-Resonance and Relaxation Enhancement (OPARACHEE) mechanism. This probe exhibited specific uptake and signal activation in neuronal cells overexpressing Cat D, which proves the feasibility of using it for *in vivo* imaging of changes in AD-related enzymatic activity.

The strategy of “drug repurposing” or “probe repurposing” has attracted attention to speed up clinical translation. Zhuang et al. successfully repurposed the classic prototype of the Aβ PET imaging probe, PiB, as a CEST probe 40. The phenolic hydroxyl group in PiB's structure is the source of exchangeable protons, giving a CEST signal at 5.8 ppm, a frequency that does not overlap with most endogenous metabolite signals. Their study demonstrated that the CEST signal intensity in the brains of APP/PS1 mice after injection is positively correlated with age (i.e., Aβ burden) and is in very good agreement with immunohistochemistry results. This work opens a new way to rapidly convert extensively preclinically and clinically validated high-affinity ligands into non-radioactive CEST probes.

Through different design strategies, CEST MRI probes have shown their value in metabolic imaging of Aβ, direct targeted imaging, monitoring enzyme activity, and rapid probe development for AD. However, this technology still has problems: most studies rely on ultra-high field strengths (7 T or 9.4 T) MRI to achieve sufficient signal-to-noise ratio, which is not compatible with mainstream clinical scanners (1.5T/3T); CEST imaging generally requires long scan times and is very sensitive to magnetic field inhomogeneity, making it prone to artifacts; the optimal dosage, long-term biosafety, and *in vivo* metabolic pathways of exogenous probes need to be systematically evaluated. Moving forward, we can enhance the role of this non-radioactive molecular imaging technology in the early diagnosis and treatment evaluation of AD by focusing on several key areas. First, we need to develop probes with higher exchange rates; second, we should continuously optimize fast CEST imaging sequences; third, we need to push forward validation research under clinical field strengths; and finally, we should explore smart, integrated CEST probes that target different pathways or mechanisms.

## 4. Summary and Perspective

Various types of probes have been developed so far. Due to their different physical and chemical properties, they are applicable to different application scenarios (**Table 5**). The development of MRI probes aimed at the Aβ species symbolizes a highly dynamic and interdisciplinary field combining expertise in chemistry, materials science, neurobiology and imaging physics. The area has undergone fundamental development and is in transition from detection of insoluble plaques to detection of soluble neurotoxic oligomers. At the same time, probe design is constantly evolving from passive imaging agents to active target probes and from single-function probes to integrated multifunctional platforms. With these joint efforts, probes in this area achieve higher sensitivity, diagnostic specificity and clinical relevance. But despite these great advances, there are still some persistent and complex challenges. The transition to clinical use of MRI probes is still difficult.

### (1) Sensitivity and signal-to-noise limits

The intrinsic low concentration of pathological aggregates of Aβ, particularly in the early stages of the disease, poses a fundamental sensitivity challenge. Traditional probes, such as gadolinium-based T1 agents or CEST probes, often produce insufficient molar signals (e.g. relaxation rate variations). To achieve a sufficient signal/noise ratio at concentrations of nanomoles or picomoles 102, although nanoparticle systems can amplify the signal through their high load, their size creates new barriers to biodistribution and target contact. To overcome this problem, it is possible to develop fast-relaxing probes with optimized geometry, to use CEST probes with multiple exchangeable sites, or to design "turn-on" probes that increase the relaxation rate upon Aβ binding, without compromising biodistribution.

### (2) High specificity and target discrimination

Highly specific expression for different Aβ species (e.g. oligomers vs. monomers or protofibrils) remains very complex. Probes dependent on general hydrophobic interactions or β-sheet recognition patterns may react with other amyloid proteins present in the brain (such as tau or α-synuclein), generating false positives 51. In addition, non-specific adsorption to non-target substances will increase background noise. Another problem with oligomer-specific probes is that the binding event itself may alter the aggregation kinetics or the toxicity profile of the oligomer and thus interfere with the pathology being measured. To overcome these challenges, probe design should use high affinity binders (e.g., peptides, aptamers) to target unique conformational epitopes. Combining orthogonal validation methods with specificity improvements (such as PEG spacers) can minimize off-target binding, while transient binding probes can reduce disturbance of oligomer kinetics.

### (3) Pharmacokinetics and *in vivo* stability

Optimal probes must have fine-tuned pharmacokinetics: effective blood-brain barrier penetration, target binding with high affinity and sufficient retention time, as well as rapid systemic clearance of unbound fractions. The balance of these competitive requirements is complex. Long-term brain retention can increase the background signal and potential toxicity, while too fast clearance can prevent sufficient accumulation of the target within the image window. In the case of metal-based probes (e.g. Gd³⁺, Mn²⁺), the *in vivo* stability of the complex is crucial to prevent the release of toxic metal ions, which may lead to nephrogenic systemic fibrosis or manganese-induced neurotoxicity 103. To overcome these problems, methods include optimizing lipophilicity and molecular weight, using prodrug strategies or receptor-mediated transcytosis for BBB penetration, and introducing rapid clearance mechanisms. For metal-based agents, macrocyclic chelators with high kinetic inertness and transmetallation resistance (e.g. DOTA) are crucial to ensure stability.

### (4) Long-term biosafety and immunogenicity

Comprehensive long-term safety is essential for all diagnostic agents intended for human use, in particular for longitudinal monitoring. In addition to concerns about heavy metal toxicity, the fate of nanomaterial-based probes requires thorough investigation. Important issues include their biodegradability, the potential toxicity of degradation byproducts, and their tendency to accumulate in organs of the mononuclear phagocyte system. In addition, both nanomaterials and biopharmaceutical probes (such as antibodies and nanobodies) pose a risk of triggering immune responses, potentially leading to hypersensitivity or accelerated blood clearance, affecting the effect of repeated dosing. To mitigate these problems, probe development should prioritize biodegradable nanomaterials with well-defined excretion pathways, surface modification with biocompatible polymers to reduce immune recognition, and immunogenicity screening of biopharmaceuticals. The "low dose, high sensitivity" design further reduces the overall material load.

This area is poised to enter a new phase characterized by intelligent design and multifunctional integration, with future breakthroughs going beyond incremental improvements to enable new diagnostic and therapeutic possibilities.

### (1) Intelligent and activatable probes

A central trend is the development of "intelligent" or activatable probes that respond to specific pathological triggers. When they encounter unique Aβ conformations or microenvironmental changes within Alzheimer’s disease plaques (for example, local variations in pH, redox state or metal ion concentration), these systems go beyond the simple "always on" contrast to turn "on" or "off" in response. Changes in the physico-chemical properties activated by these targets (such as aggregation or rotation time) can generate a significant and specific increase in MRI signals (e.g., relaxation rate or CEST effect), significantly improving the contrast-to-noise ratio 14.

### (2) Multimodal integration

Integrating complementary imaging modes on a single probe platform is a powerful strategy to overcome the inherent limitations of individual technologies. An integrated probe compatible with MRI, PET and optical imaging would allow, after preliminary screening based on the high sensitivity and quantitative capacity of PET, anatomical localization and longitudinal tracking thanks to the superior spatial resolution and tissue contrast of MRI, as well as surgical guidance or histopathological validation by the high molecular sensitivity of fluorescence.

### (3) Probe design and image analysis driven by artificial intelligence

AI and machine learning will greatly accelerate this progress. Computer simulation methods, including virtual screening and generative models, will accelerate the optimization of affinity, selectivity and blood-brain barrier (BBB) penetration of new targeted ligands. In addition, AI- based image analysis algorithms can extract fine multidimensional attributes from image data, allowing automated, accurate and reproducible segmentation and quantification of Aβ pathology, reducing inter-observer variability and improving diagnostic reliability.

### (4) Transition to theranostic platforms

The ultimate progress is to develop true theranostics combining diagnosis and therapy. The new generation of probes could not only serve as imaging biomarkers but also as therapeutic vectors. Once specifically targeting Aβ aggregates, these platforms could directly neutralize toxic oligomers via their functional components, effectively administer targeted therapeutic loads (such as anti-aggregation compounds or immunomodulators), or even promote clearance mechanisms. This paradigm will allow disease detection and targeted interference simultaneously, paving the way for personalized and precision treatment for Alzheimer’s disease.

### (5) Regulatory compliance and standardization

When introducing new Aβ-targeted MRI probes into the clinic, the regulatory criteria established by the agencies must be met. For example, the FDA requires that new diagnostic agents ensure clearly defined sensitivity and specific thresholds and consistent performance between batches. On the other hand, the guidelines for the biosafety assessment of nanomaterials drawn up by the European Medicines Agency (EMA) emphasise the importance of assessing the long-term toxicity of new diagnostic agents and their metabolic pathways. To meet these basic requirements, it is necessary to build a standardized image data analysis workflow to facilitate the transition to clinical applications on a larger scale.

## Figures and Tables

**Table 1:** Summary of Small Molecule MRI Probes for Aβ Imaging.

Probe	Type	Targeting Group	Signal Unit	Relaxivity	Binding Constant	Imaging Model	Advantages	Ref.
DOTA-Lys₃-BTA	T1	Benzothiazole	Gd-DOTA	r1=8.6 mM⁻¹ s⁻¹	K_i_=71 nM	*In vitro* Aβ₁₋₄₂ aggregates	Positively charged linker enhances binding affinity by 3-fold	57
LCO-DOTA-Gd	T1	Luminescent conjugated oligothiophene (LCO)	Gd-DOTA	Not specified	Not specified	28-month-old Tg-APPSwe mice	Integrated targeting and BBB penetration, long-lasting imaging	45
R3VQ-S-(DOTA/Gd)_3_	T1	Camelid single-domain antibody (VHH)	Gd-DOTA	r1=35 mM⁻¹ s⁻¹	IC_50_=19 nM	15-month-old PS2APP mice	10-fold higher relaxivity than clinical agents, BBB penetration	58
Gd(DO3A-PiB)	T1	PiB	Gd-DO3A	8.1 mM⁻¹s⁻¹	K_d_=180±10 μM	Human AD brain tissue sections	Environmentally responsive relaxivity enhancement	34
GdDOTA-ob5	T1	ob5 (DNA aptamer)	Gd-DOTA	r1=28 mM⁻¹ s⁻¹	Not specified	3xTg-AD mice (5-8 months)	Exceptional oligomer selectivity	32
MnL3	T1/T2	Tryptamine	Mn(II) complex	r1=7.26, r2 = 23.26 mM⁻¹ s⁻¹	K_d_=3.19 μM	*In vitro* Aβ fibrils	Simultaneous T1 and T2 imaging, Potential safety of endogenous metal	61
FSB	¹⁹F	Bis-styrylbenzene	¹⁹F (1 F)	Not specified	Not specified	Tg2576 mice	First ¹⁹F probe, proof-of-concept	62
TFMB-3Et	¹⁹F	Benzoxazole	-OCF₃ (3 F)	Not specified	Not specified	APP-transgenic mouse	Hydrophilicity, cross the BBB and bind to senile plaques	63
Shiga-Y5 (FMeC1)	¹⁹F	Curcumin	-OCF₃ (6 F)	Not specified	Not specified	Tg2576 mice	Keto-enol tautomerism enables dynamic binding regulation	64
Shiga-X22 (XP7)	¹⁹F	Benzoxazole	-OCF₂CF₃ ()	Not specified	Not specified	APP/PS1 mice	PEG overcomes post-binding signal, 100% *in vivo* specificity	65
Shiga-Y51	¹⁹F	Curcumin	-OCF₃ (6 F)	Not specified	Not specified	APP/PS1 mice	First oligomer-selective ¹⁹F probe	39
Compound 7d	¹⁹F	Indanone	Multi-fluorine	Not specified	K_d_=367-384 nM	ICR mice	Low toxicity (LD₅₀ > 50 mg/kg), rapid brain uptake and clearance	19
Curc-Glu-F9	¹⁹F	Curcumin	Perfluoro-tert-butyl (9 F)	T1=558 ms, T2=139 ms	K_a_=1.1 × 10⁵ M^-1^	*In vitro* Aβ oligomers	Distinguishes oligomers from fibrils, high fluorine content	66

**Table 2:** Comparison of PET and MRI Probes Sharing the Same Targeting Motif.

PET Prototype	MRI Adapted Version	Key Modifications	Key Performance Changes	Ref.
[¹¹C]PiB	Gd(DO3A-PiB)	¹¹C to Gd-DO3A; trilysine linker	Dose: 10-20 μCi/mouse vs 0.1-0.2 mmol Gd/kg Radiation: Ionizing vs None Sensitivity: ~nmol/L vs ~pmol/L (2-4× relaxivity)	45,46
[¹⁸F]DDNP	DDNP-SPIO	¹⁸F removed; PEG-PLA coating	Dose: 15-25 μCi/mouse vs 0.3-0.5 mg Fe/kg Radiation: Ionizing vs None Sensitivity: 3-5 mm vs ~100 μm (r₂ = 140.6 mM⁻¹s⁻¹)	69, 70
[¹⁸F]AV-45	ADx-001 (liposomal Gd-DOTA)	¹⁸F → Gd-DOTA; DSPE-PEG-styryl-pyrimidine	Dose: 20-30 μCi/mouse vs 0.15-0.25 mmol Gd/kg Radiation: Ionizing vs None Sensitivity: ±15% error vs ±8% (r₁ ≈ 31 mM⁻¹s⁻¹; sensitivity >80%)	26, 59
[¹⁸F]FMeC1	FMeC1-¹⁹F probe	¹⁸F removed; 7-unit PEG spacer	Dose: 15-20 μCi/mouse vs 100-200 mg/kg Radiation: Ionizing vs None Sensitivity: Fibrils only vs Oligomers/fibrils (4× SNR)	38, 66

**Table 3:** Summary of Nanoparticle-Based MRI Probe for Aβ Imaging.

Probe	Type	Size (nm)	Targeting Mechanism	Signal Unit	Relaxivity	Animal Model	Key Advantages	Ref.
Cur-MNPs	T2	~10–15	Curcumin	SPIONs	Not specified	Tg2576 mice	Natural targeting moiety, good biocompatibility	69
PiB-Mn-Zn Ferrite	T2	~20	PiB	Mn-Zn ferrite	r2=169.93 mM⁻¹s⁻¹	Not specified	Ultra-high r2 relaxivity	71
W20/XD4-SPIONs	T2	~50	Anti-Aβ oligomer scFv	SPIONs	Not specified	APP/PS1 mice	Simultaneous imaging and clearance of oligomers	72
Anti-Aβ Antibody-SPIONs	T2	~15–20	Anti-Aβ antibody	SPIONs	Not specified	APP/PS1 mice	High specificity, no BBB opening required	73
NP@angiopep-2/CD137	T2	~100	Angiopep-2 + CD137 antibody	SPIONs	Not specified	APP/PS1 mice	Targets neuroinflammation	74
aAβ-BTRA-NC	T1	~100–150	Anti-Aβ antibody	Gd-DTPA	Not specified	Tg mice of different ages	Active BBB targeting	75
UFNPs@PEG/PZD	T1-T2	<15	Phenothiazine	Ultrasmall ferrite	Not specified	APP/PS1 mice	Complementary signals improve accuracy; ultra-small size facilitates passive diffusion	76
RVG29-Rifampicin-Gd NPs	T1	~120	RVG29	Gd	Not specified	AD model mice	Active targeting + drug delivery	77
CR-BSA-(Gd-DTPA)n	T1	~50–80	Congo red	Gd-DTPA	Not specified	APP/PS1 mice	Specific Aβ fibril binding	78
ADx-001 (Liposome)	T1	~140	ET3-73	Gd-DOTA	Not specified	APP/PS1 mice	100% imaging specificity, low toxicity	60
PMLA-CUR-Gd-DOTA	T1	~50–100	Curcumin	Gd-DOTA	r1=12.5 mM⁻¹s⁻¹	Human AD brain sections + AD mice	Targets Aβ plaques, polymer carrier improves pharmacokinetics	59

**Table 4:** Summary of CEST and Multimodal probes for Aβ Imaging.

Probe	Type	Targeting Mechanism	Signal Unit	Signal Property	Animal Model	Key Advantages	Ref.
Angiopep-2	CEST	LRP1-targeting peptide	Amide/hydroxyl protons	CEST signal at 3.2 ppm	APP/PS1 mice	Metal-free, active BBB penetration, consistent with immunohistochemistry	41
PiB Derivative	CEST	Phenolic hydroxyl as exchangeable proton source	Hydroxyl protons	CEST signal at 5.8 ppm	APP/PS1 mice	Rapid conversion from PET probe, radiation-free	103
2-Deoxy-D-glucose (2DG)	CEST	Brain glucose metabolism	Hydroxyl protons	GlucoCEST effect	AD model mice	Endogenous, no exogenous substance, assesses brain metabolism	100
NP@SiO₂@F-SLOH	MRI/FL	F-SLOH selective for Aβ oligomers	NaGdF₄ + Fluorescence	T1 enhancement + fluorescence signal	11-month-old AD mice	Simultaneous imaging and inhibition of Aβ fibrillation, ROS reduction	92
Dyad-3	MRI/FL	Cyanine scaffold	Gd(DOTA) + NIR fluorescence	T1 enhancement + NIR fluorescence	12-month-old 5XFAD mice	Integrated small-molecule design, inhibits Aβ fibrillation	52
BSA@FGQDs	¹⁹F MRI/FL	Bovine serum albumin + fluorinated graphene quantum dots	¹⁹F + Fluorescence	Fluorescence quenching + ¹⁹F signal activation	AD model mice	Completely metal-free, complementary dual-modal signals	95
Gd-NP@SiO₂@HY5	MRI/FL	HY5 specific for Aβ₄₂ + H₂O₂ responsive	NaGdF₄ + NIR fluorescence	T1 enhancement + ratiometric fluorescence	APP/PS1 mice	Dual-responsive (Aβ + ROS), oxidative stress monitoring	91
NaGdF₄-PMPC	SWI MRI	PMPC mimics acetylcholine targeting α7 nAChR	NaGdF₄	Delayed enhancement, persists for 24 h	Early-stage AD model mice	Targets neuroinflammation, ultra-small size facilitates diffusion	93

**Table 5:** Comparison of Key Features, Advantages, and Limitations of Different Types Aβ-Targeted MRI Probe.

Probe Type	Signal Mechanism	Targeting Strategy	BBB Penetration	Key Advantages	Key Limitations
Small-Molecule T1 Probes	T1-weighted positive contrast	Small-molecule ligands (PET-derived, aptamers, nanobodies)	Physicochemical optimization	Well-defined structure, high designability, exceptionally high relaxivity	Low BBB efficiency, metal toxicity concerns
¹⁹F Probes	Direct ¹⁹F imaging (zero background)	Benzoxazole/curcumin derivatives, conformation-locking	Hydrophilicity optimization	No endogenous background, aggregation state selectivity	Low sensitivity, high dose, signal quenching
T2 Nanoparticle Probes	T2/T2*-weighted negative contrast	Antibodies, peptides, small-molecule ligands	Active targeting, pathological BBB leakage	High sensitivity, multifunctional surface, theranostic potential	Negative contrast confusable with hemorrhage, safety concerns
T1 Nanoparticle Probes	T1-weighted positive contrast	Antibodies, peptides, active targeting ligands	Receptor-mediated transcytosis, passive diffusion	Clear positive contrast, dual-modality imaging capability	Size-dependent BBB penetration, metal retention risk
CEST Probes	Chemical exchange saturation transfer	Targeting peptides, PET probe repurposing, endogenous metabolites	Receptor-mediated transcytosis, physicochemical optimization	Metal-free, endogenous analogs, large design space	Low sensitivity, long scan time, requires ultra-high field
Multimodal Probes	MRI + FL/¹⁹F/PET	Integrated multiple targeting functions	Combined passive, active, and physical strategies	Complementary signals, functional integration, theranostic potential	Complex design, unclear metabolism
